# Evaluating the Accuracy of the QCEIMS Approach for Computational Prediction of Electron Ionization Mass Spectra of Purines and Pyrimidines

**DOI:** 10.3390/metabo12010068

**Published:** 2022-01-12

**Authors:** Jesi Lee, Tobias Kind, Dean Joseph Tantillo, Lee-Ping Wang, Oliver Fiehn

**Affiliations:** 1 Department of Chemistry, University of California, Davis, CA 95616, USA; jselee@ucdavis.edu (J.L.); djtantillo@ucdavis.edu (D.J.T.); 2 West Coast Metabolomics Center, University of California, Davis, CA 95616, USA; tkind@ucdavis.edu

**Keywords:** quantum chemistry, in silico prediction, mass spectra, QCEIMS

## Abstract

Mass spectrometry is the most commonly used method for compound annotation in metabolomics. However, most mass spectra in untargeted assays cannot be annotated with specific compound structures because reference mass spectral libraries are far smaller than the complement of known molecules. Theoretically predicted mass spectra might be used as a substitute for experimental spectra especially for compounds that are not commercially available. For example, the Quantum Chemistry Electron Ionization Mass Spectra (QCEIMS) method can predict 70 eV electron ionization mass spectra from any given input molecular structure. In this work, we investigated the accuracy of QCEIMS predictions of electron ionization (EI) mass spectra for 80 purine and pyrimidine derivatives in comparison to experimental data in the NIST 17 database. Similarity scores between every pair of predicted and experimental spectra revealed that 45% of the compounds were found as the correct top hit when QCEIMS predicted spectra were matched against the NIST17 library of >267,000 EI spectra, and 74% of the compounds were found within the top 10 hits. We then investigated the impact of matching, missing, and additional fragment ions in predicted EI mass spectra versus ion abundances in MS similarity scores. We further include detailed studies of fragmentation pathways such as retro Diels–Alder reactions to predict neutral losses of (iso)cyanic acid, hydrogen cyanide, or cyanamide in the mass spectra of purines and pyrimidines. We describe how trends in prediction accuracy correlate with the chemistry of the input compounds to better understand how mechanisms of QCEIMS predictions could be improved in future developments. We conclude that QCEIMS is useful for generating large-scale predicted mass spectral libraries for identification of compounds that are absent from experimental libraries and that are not commercially available.

## 1. Introduction

Purines, pyrimidines, and their derivatives are critically important biochemicals to sustain life. For example, adenine, cytosine, thymine, and guanine serve as building blocks for DNA and RNA. Many derivatives, including methylpurines and methylpyrimidines, but also hypoxanthine, xanthine, or pteridine derivatives, serve essential functions in multiple metabolic pathways and extracellular processes. In humans, enzymatic pathways involving the metabolism of purines and pyrimidines are key biological pharmaceutical targets, for example, for anticancer [1,2,3], antiviral [4,5,6], antimicrobial [7,8] and anti-inflammatory [9,10] purposes. The metabolites themselves are often used as biomarkers for identifying cancer risk [11] and detecting oxidative DNA damage [12]. Given their critical roles in biological systems, further modifications of purines and pyrimidines might exist for which no chemical standards are available. To identify such molecules by mass spectrometry (MS), reference spectral libraries must be extended to enable computational generation of putative structures, followed by accurate in silico MS spectra predictions. However, reference databases are difficult to curate because the spectra are highly sensitive to the type of MS machine used. Less than 1% of all known small compounds covered in databases such as PubChem have experimental mass spectra available in licensed libraries such as Wiley or National Institute of Standards (NIST) [13,14,15] or public reference databases such as MassBank of North America (MassBank.us), covering today over 650,000 spectra [16]. Utilizing ab initio-generated in silico spectra may help fill those gaps.

While rule-based [17,18,19] or machine-learning [20,21] approaches have been tested extensively for computational generation of MS libraries, ab initio quantum chemistry approaches have been made available more recently [22,23,24,25]. The Grimme group developed the Quantum Chemical Electron Impact Mass Spectrum (QCEIMS) program that utilizes Born–Oppenheimer molecular dynamics (BOMD) coupled with Boltzmann statistics and isotopic distribution [23,26]. Briefly, QCEIMS samples the ground-state molecular geometry and then calculates the molecular dynamics (MD) trajectories for 70 eV excited states over sub-picosecond time scales while recording specific fragmentation reactions. The statistics of many fragmentation trajectories are then summarized to yield a list of fragment ions and their associated intensities [24]. While much theoretical work on QCEIMS has been published in the past [27,28,29,30,31,32,33,34], currently only four purines and pyrimidines have been investigated in detail [27]. Density function theory (DFT) has also been used for calculating the partition coefficients in organic solvents [35]. Here we extended the work on QCEIMS to cover many more systems, resulting in an in silico electron ionization (EI) mass spectral library for 80 different purine and pyrimidine derivatives by QCEIMS calculations. To examine the accuracy and usability of such QCEIMS predicted spectra, we compared these standard compounds to existing experimental 70 eV EI mass spectra from the National Institute of Standards (NIST 17) database. We further investigated the relationship of structural characteristics to the accuracy of QCEIMS prediction within these classes of particular biomolecules.

## 2. Results

**Figure 1:**
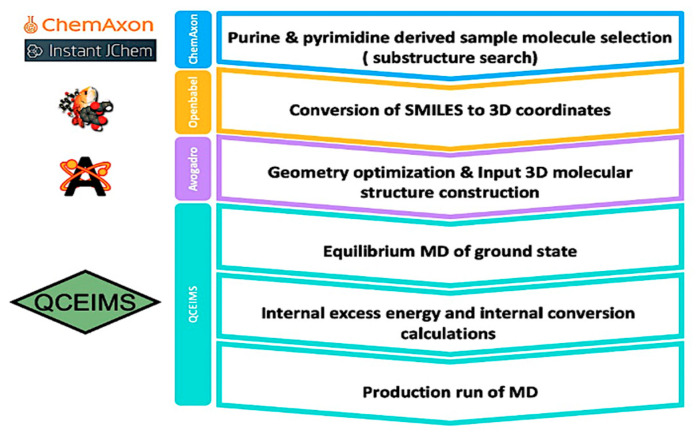
Overall workflow from selection of molecules to generating QCEIMS mass spectra.

The workflow of this study is depicted in Figure 1. Details are given in the methods section.

To investigate the accuracy of QCEIMS calculations, we generated in silico EI mass spectra of 80 compounds (14 pyrimidines and 66 purines) and compared these to experimental EI mass spectra using mass-weighted dot product (Wdot) similarity scores. QCEIMS calculations were computationally intensive but could be performed on modern hardware for tens to hundreds of small molecules in reasonable overall time. One of the main factors that affects the computational costs using QCEIMS is the size of the molecules, because the individual simulations scale is O(N^2^) to O(N^3^) with basis set size [36], and the number of simulations is proportional to the number of atoms, making the overall scaling O(N^3^) to O(N^4^) with respect to molecular size. In the dissociation MD step, the software runs 25 trajectories per atom. We previously found that the default number is reasonable for estimating the frequency of reactions occurring at a given internal excess energy and yields good ion statistics mimicking experimental mass spectra [34]. Our pyrimidines and purines ranged in molecular size from 12 to 22 atoms. For the 80 molecules examined here, 3293 CPU hours were used in total, with an average of 41.6 h per mass spectrum (Figure S1).

We first evaluated the quality of generated QCEIMS spectra on three levels: (a) overall scores and scores in different purine and pyrimidine subclasses, (b) accuracy in identifying the correct molecule when searching the QCEIMS spectra against the large NIST 17 library of over 267,000 molecules, (c) analyzing the number of ions that were found in both experimental spectra and in QCEIMS spectra versus ions that were only detected in experimental or in QCEIMS spectra. From this data, it became clear that QCEIMS still underperforms in specific fragmentation reactions with ions that were either observed in the experiment but not predicted, or vice versa. Hence, we finally investigated in detail the trajectories of typical neutral losses and retro Diels–Alder reactions that are typically observed for purines and pyrimidines to better understand how to improve QCEIMS.

### 2.1. Overall Similarity Scores and Investigation of Structural Subclasses

**Figure 2:**
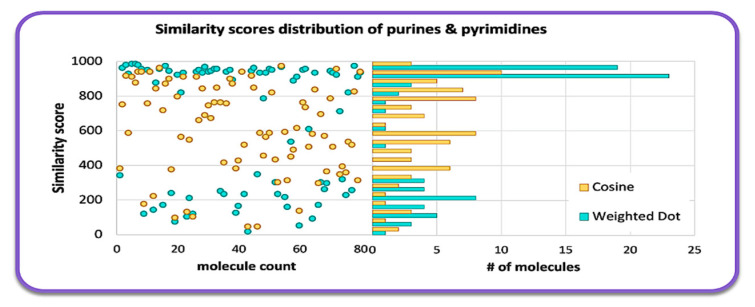
Histogram of MS similarity of QCEIMS predicted versus experimental mass spectra for 80 purines and pyrimidines, comparing weighted dot product and cosine similarity scores.

**Figure 3:**
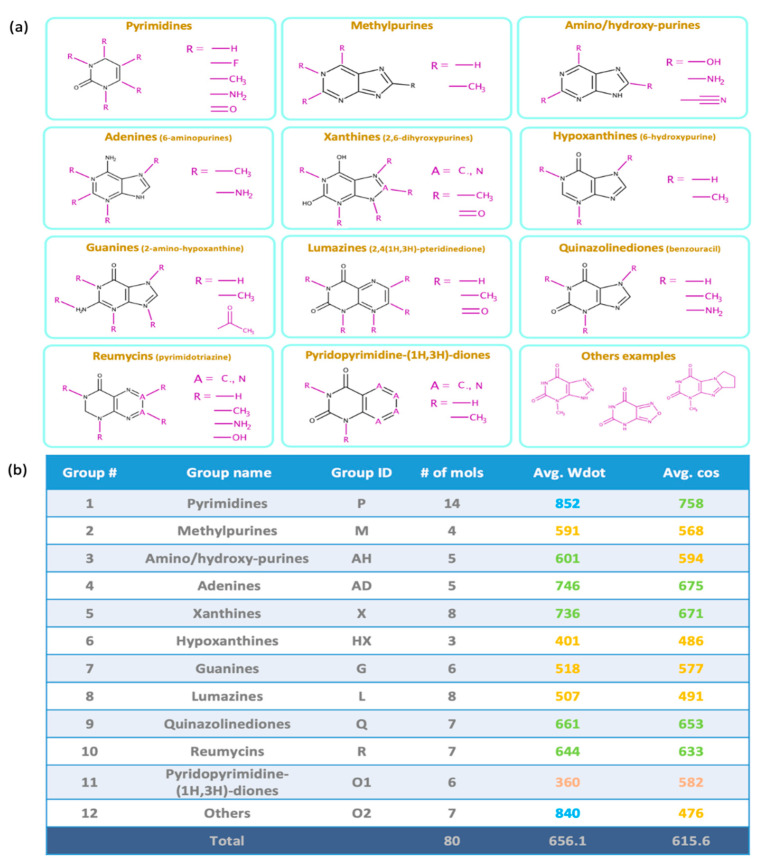
(**a**) Categorization of 80 purines and pyrimidines by structural subclasses. (**b**) MS similarity purine and pyrimidine subclasses matching QCEIMS predicted spectra against experimental spectra. Colors indicate excellent matches (>800, blue), good (>600, green), fair (>400, yellow) and poor (<400, red) similarity scores.

For over 25 years, mass-weighted dot product (Wdot) scores have been used to quantitatively evaluate the similarity of experimental against library mass spectra for small molecules [15], usually outperforming simple cosine scores. Wdot scores above 850 are considered good matches whereas those below 600 are characterized as poor matches. Across all 80 tested molecules, we found an average Wdot score of 656 for the QCEIMS predicted spectra. This overall acceptable score, however, showed large ranges in quality, ranging from scores as low as 100 to a nearly perfect score of 978 (Figure 2). Indeed, the Wdot scores showed a bimodal distribution, whereas the simple cosine scores were more equally distributed across poor and high quality scores (Figure 2). To investigate if substructures were significantly impacting overall Wdot scores and to explain especially the bimodal distribution, we manually categorized the purine and pyrimidine derivatives into 12 groups (Figure 3b). Purines were classified into adenines (6-aminopurines), or xanthines (2,6-dihydroxypurines) with additional functional groups such as methyl-, amino-, hydroxy-, or aza-derivatives (Figure 3a). Pyrimidines showed the highest average Wdot scores, possibly impacted by their smaller molecular size compared to others. Next, adenines and xanthines demonstrated high average similarity scores, while hypoxanthines, lumazines, guanines, and methylpurines showed comparatively lower scores. Yet even within these subgroups the similarity scores often varied widely and were not clearly related to particular substructure motifs.

**Figure 4:**
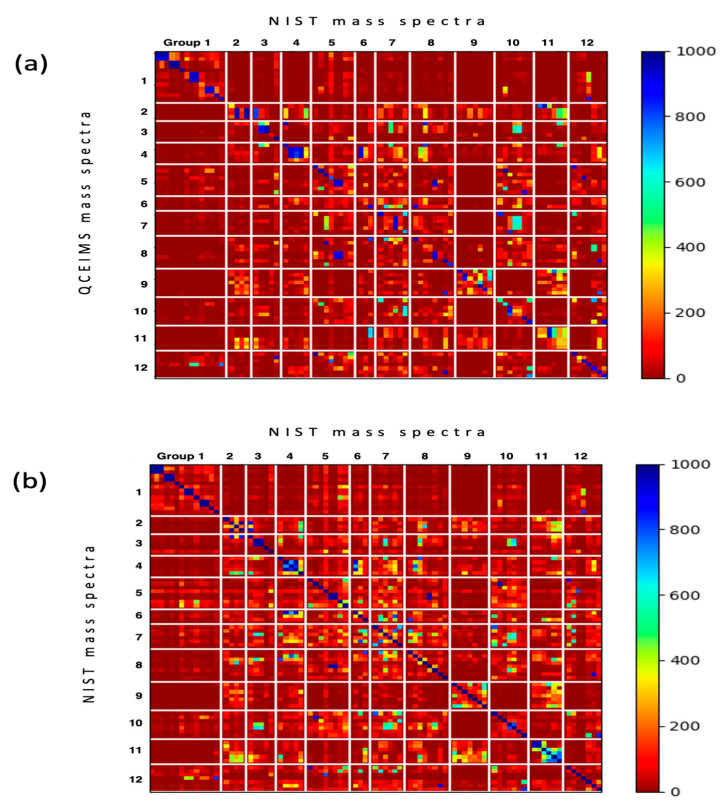
Heatmaps of weighted dot product similarity scored mass spectra sorted by the 12 subclasses of purines and pyrimidines. (**a**) Matching the 80 QCEIMS predicted versus all experimental mass spectra in the NIST 17 library. (**b**) Matching the target 80 experimental NIST 17 spectra of purines and pyrimidines versus all experimental mass spectra in the NIST 17 library.

Next, we constructed similarity score matrices of all 80 molecules to examine pairwise molecule relationships, comparing both predicted and experimental spectra. All similarity scores are given in Table S1. The similarity scores were computed for all pairs of 80 input molecules. Diagonal cells directly give the similarity scores matching in silico spectra to NIST 17 experimental spectra for each molecule (Figure 4a), with the best scores labeled blue and poorest scores labeled red. All other cells represent comparisons to other molecules. Many direct QCEIMS/NIST 17 comparisons were indeed found as the highest similarity scores in each row. Hence, if such spectra were not already present in the NIST 17 library, generating QCEIMS spectra would have been useful for successful identifications in untargeted metabolomics. However, some subclasses like lumazines (group 8) produced QCEIMS spectra that showed highest Wdot scores to compounds of another subclass, here xanthines (group 5), as indicated by a blue block of similarity scores (Figure 4a). In addition, some compounds in a specific subgroup showed higher Wdot scores to other compounds in the same subgroup, for example, for methylpurines (group 2, Figure 4a). Yet a simple comparison of NIST 17/NIST 17 spectra (Figure 4b) showed that many experimental spectra also showed high similarity scores to compounds of the same structure subgroup or even other structure subgroups. Indeed, the same Wdot score matrices were found in the NIST 17/NIST 17 matrix (Figure 4b) as in the QCEIMS/NIST 17 matrix (Figure 4a), for example, between subgroups (hypoxanthines versus guanines, i.e., group 6 versus 7, Figure 4b) and within subgroups (methylpurines group 2, amino/hydroxy-purines group 3). A substantial number of off-diagonal similarity scores in the NIST17/NIST 17 matrix (Figure 4b) were found at Wdot scores >600 (light to dark blue colors), showing that experimental GC-MS studies also face the possibility of misidentifications due to the high number of structurally similar compounds. Hence, even if QCEIMS calculated spectra were almost identical to the corresponding experimental ones, the two matrices in Figure 4a,b would likely be indistinguishable. Hence, the fact that these two matrices show remarkable resemblances showcases the ability of QCEIMS to accurately predict mass spectra.

### 2.2. Validating QCEIMS Spectra by Matching against Large Libraries

We further validated the quality of our QCEIMS spectra by comparing the in silico spectra against all compounds in large databases. This validation is similar to researchers matching experimental data against libraries to identify compounds in untargeted analyses. Ideally, the in silico spectra will give the correct structure as the top hit in library searches, similar to the use of libraries against experimental spectra. However, as we showed above, the large number of structurally similar compounds may lead to lower score accuracies. Researchers will usually stop searching below the top 10 matching hits based on practicality [37,38,39] Therefore, we used the same schema here to evaluate the practical quality of our 80 QCEIMS spectra of purines and pyrimidines. Of 80 QCEIMS spectra 36 indeed ranked as top hits when searching the spectra against the NIST 17 library of more than 267,000 spectra in total (Table 1). Of our QCEIMS spectra 58% ranked within the top 3 matched molecules, and 74% of our in silico spectra identified the correct molecule within the top 10 hits (Table 1). These results showed that QCEIMS spectra can be useful in experimental settings, but not in all cases. Interestingly, 11 QCEIMS spectra were not even matched within the top 100 compound hits when searched for in the NIST 17 library. Examples of good hits within the top 3 scores are 6-methyluracil, 2,6-dihydroxy-7-methylpurine, and 3,5,7-trimethyl-5H-pyrimido [4,5,-E] [1,2,4] triazine-6,8-dione, whereas adenine, 2-purinol, and pyrido[4,3-d]pyrimidine-2,4(1H,3H)-dione were found at lower ranks than the top 10 hits (Table S1). When we compared these three poor performing examples, we found that Wdot score similarities were reasonably high, ranging between 874 and 940. The failure in assigning the correct structures to these QCEIMS spectra was a result of the large number of molecules with even higher Wdot scores owing to high structural similarity with the target compounds. Conversely, even for some of the excellent top-scoring hits, for instance, 6-cyanopurine, 2-aminopurine, and 7-methylguanine, we found low Wdot similarity scores, <600, of only 73, 139, and 127. Hence, the usability of QCEIMS spectra is not only dependent on the overall achieved Wdot scores when matched to experimental spectra but also related to the uniqueness of the spectra themselves as well as the investigated chemical structures being distinguishable from other molecules within a mass spectral library or an experimental GC-MS sample.

### 2.3. Matching, Missing, Extra Fragment Ions Versus Ion Abundances

**Figure 5:**
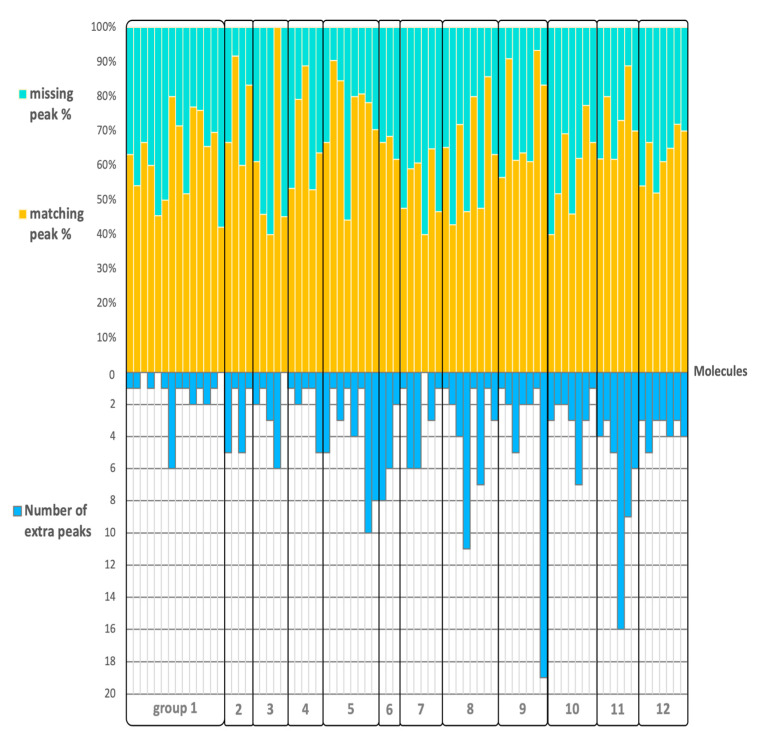
Detailing the accuracy of QCEIMS predictions of 80 purines and pyrimidines sorted by the 12 subclasses. **Top**: percentage of the number of matching and missing fragment ions. **Bottom**: number of additionally predicted fragment ions when comparing to NIST 17 experimental mass spectra.

This validation of QCEIMS spectra was based on classic Wdot similarity scores that combine information from the presence and abundance of ions across a spectrum. We therefore continued our assessment of the quality of QCEIMS spectra by computing the Jaccard index, which essentially is the ratio of intersection and union of two different data sets and allows estimating the similarity of two data sets. When the Jaccard index is 1, the two data sets are identical, and when it is 0, they are completely different. The Jaccard index was calculated for each molecule to determine the percentage of matching and missing peaks as well as extra fragment ions that were generated by the QCEIMS process but that were not found in the experimental spectra. We used a 2% cut-off threshold for experimental fragment ions to account for the problem of correctly finding low-probability reactions in the statistics of QCEIMS trajectories. On average, 65% of all fragment ions in experimental spectra were correctly predicted by QCEIMS, ranging from 40 to 100% for different precursor ions (Figure 5). We did not observe a clear pattern for the percentage of correctly predicted fragment ions or the number of additional QCEIMS fragment ions based on our structure classification. When we increased the threshold to a 20% ion abundance cut-off, we found an expected increase in the number of correctly predicted ions to an average of 88%, but again without a preference for one compound class over another (Figure S2). This high number of correctly predicted ions meant that, for the most part, QCEIMS trajectories correctly reflected experimentally observed fragmentation reactions, especially for reactions that were frequently found and therefore led to abundant ions. When calculating the Jaccard similarity score for all compounds and testing the Spearman-rank correlation of the Jaccard similarity to overall Wdot or cosine similarity scores, we found significant trends at *r* = 0.33 with *p* < 0.0025 for Wdot and *r* = 0.30 with *p* < 0.0065 for cosine similarity. However, we did not find a correlation of Jaccard scores to the hit-rank when comparing QCEIMS spectra to overall NIST 17 searches as given above. We propose that this is because the Jaccard scores do not take ion abundances into account. Jaccard similarity scores ranged between 0.27 to 0.88 but were not found to be statistically different between structure classes. This finding supported the notion that accuracy of QCEIMS predictions did not depend on structural similarities of molecules (at least, according to conventional classification), but instead, that the accuracy of QCEIMS predictions must be accounted for by differences in the simulation trajectories that were specific to each individual molecule. We therefore continued detailed investigations into fragmentation reactions for specific pathways across molecules.

### 2.4. Detailed Studies of Fragmentation Pathways

#### 2.4.1. Abundance of Molecular Ions in QCEIMS Predictions

Weighted dot product similarity scores (Wdot) place higher weights on large *m/z* values than on lower *m/z* values in mass spectral comparisons. Hence, Wdot scores are highly impacted by the abundance of molecular ions in QCEIMS predictions. As we saw a large discrepancy between Wdot and cosine similarities, we first investigated the fraction of molecular ions that did not fragment under the QCEIMS conditions studied here. In principle, aromatic molecules should show less fragmentation compared to aliphatic molecules because of the well-known stabilizing effect of aromaticity. All purine and pyrimidine molecules investigated here therefore shared this high probability of molecular ion survival, owing to the delocalized π electron system of the pyrimidine and imidazole rings, ranging from 0 to 100% intensity. Indeed, for all 80 molecules, NIST 17 experimental spectra presented molecular ions, and theoretical QCEIMS spectra correctly predicted the presence of all 80 molecular ion peaks. Experimentally, for 61 molecules, molecular ions were found as the most abundant (base peak) ions in the electron ionization mass spectra. However, only for 42 of these molecules was the molecular ion abundance correctly predicted by QCEIMS (within ±2%), including 23 molecules that were correctly predicted with molecular ions at base peak intensity. When we increased the window of predicted accuracy to 10%, we found an increase to 46 out of 80 molecules with accurately predicted molecular ion intensities. The molecular ion abundances of methylpurines, amino- and hydroxypurines, adenines, and guanines were accurately predicted by QCEIMS as base peak ions. For 24 molecular ions, intensities were underestimated by an average of 33% (ranging from 3 to 79, Table 2), 12 molecular ion peaks were overestimated by QCEIMS by an average of 40% difference (ranging from 6 to 98%, Table 2). Surprisingly, although pyrimidines showed the highest average similarity score of all tested structure subgroups (group 1 in Figure 3), for half of the tested pyrimidines the molecular ion peak abundance was underestimated, and only one was overestimated, meaning that for pyrimidines, other fragment ions were predicted remarkably well, unlike for other substructure classes. In contrast, lumazines had the worst Wdot scores when comparing QCEIMS predicted to NIST 17 experimental spectra (group 8 in Figure 3), possibly because four of the eight lumazine compounds also performed very poorly in the QCEIMS prediction of the intensity of molecular ions (Table 2). These examples showed that prediction of the abundance of molecular ions was not the only cause of poor Wdot similarity scores. To further investigate details of the reasons for poor mass spectral predictions in QCEIMS of purines and pyrimidines, we investigated specific reactions across all molecules in detail.

#### 2.4.2. Retro Diels–Alder Reaction and Loss of Isocyanic- or Cyanic Acid (NCOH, HNCO, 43 u)

**Figure 6:**
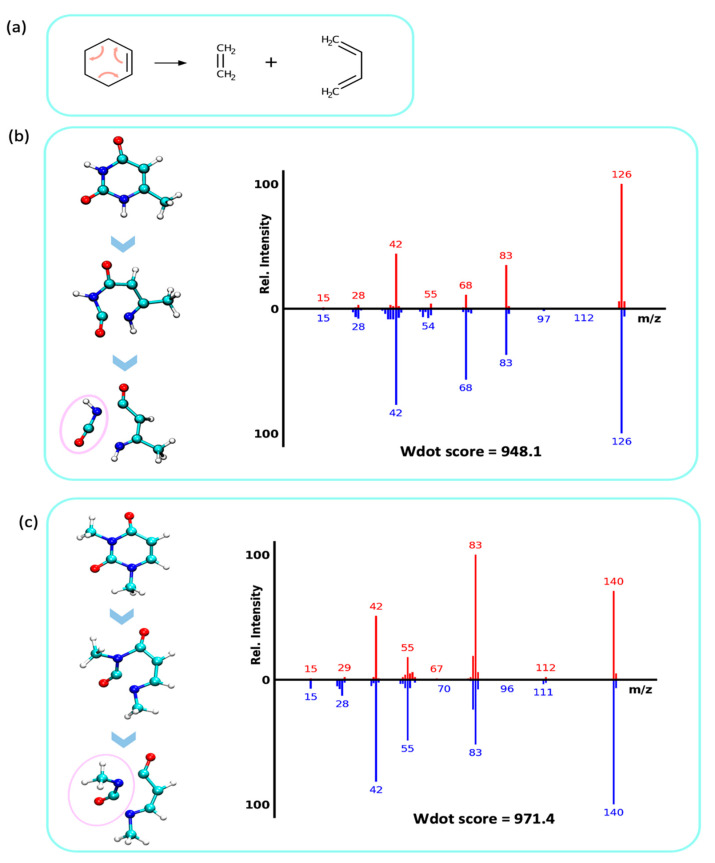
Retro Diels–Alder reactions in electron ionization fragmentation. Top: QCEIMS, bottom: experimental spectra. (**a**) Mechanistic example of a retro Diels–Alder fragmentation. (**b**) Analysis of QCEIMS trajectories for 6-methyluracil marking the neutral loss of isocyanic acid (43 u, pink circle, yielding *m/z* 83). (**c**) Analysis of QCEIMS trajectories for 1,3-dimethyluracil marking the neutral loss of methyl-isocyanic acid (57 u, pink circle, yielding *m/z* 83).

One of the most prominent fragmentation reactions in purine and pyrimidine molecules is the retro Diels–Alder reaction (rDA) reaction, a well-known concerted rearrangement type of fragmentation mechanism. Here, the cyclohexene unit-like molecules fragment to form diene and dienophile (*ene*) products (Figure 6a). In the MD trajectories, we observed the pyrimidine ring undergoing rDA fragmentation with both σ-bonds breaking on the fs time scale, which essentially is a concerted step as previously studied [27]. First, the N1–C2 bond was broken to open the ring, followed by a N3–C4 bond breakage (atom positions as numbered in Figure 6b) that resulted in neutral losses of either cyanic acid (N≡COH, 43 u) or isocyanic acid (HN=C=O, 43 u). This neutral loss of 43 u yielded ion signals at *m/z* 83, 83, 84, 87, 98 in the mass spectra of molecules 1- and 6-methyluracil (Figure 6b; spectra P1; P2 in Figure S3). The same rDA and HNCO losses were observed for molecules associated with spectra P3, P4, and P7 (Figure S3). In comparison, 3,6-dimethyluracil and 1,3-dimethyluracil contained an additional methyl-group at the N3 position. Consequently, the QCEIMS trajectories led to analogous rDA reactions but lost methyl-isocyanate (Figure 6c, CH3-N=C=O, 57 u) instead of HNCO (spectra P5, P6, Figure S3).

**Figure 7:**
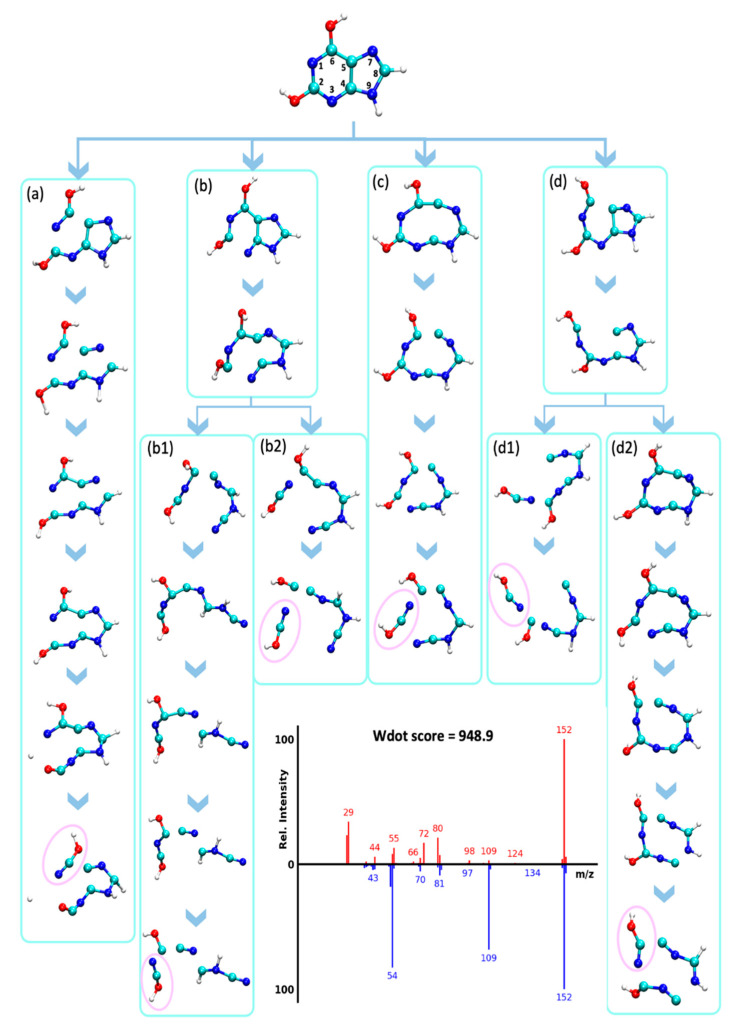
Analysis of QCEIMS trajectories for xanthine marking multiple dissociation pathways (**a**–**d**) to lose isocyanic- or cyanic acid (HNCO/NCOH, 43 u, noted by pink circles). Note: none of the trajectories predicted the direct neutral loss of 43 u from the molecular ion to yield *m/z* 109. Mass spectrum insert: **Top**: QCEIMS, **bottom**: experimental spectrum.

The rDA reaction pathway was previously reported for EI-MS spectra of purines like xanthine (X1) [40] and uric acid (X4) [41]. Our MD simulations confirmed that the rDA reaction opens the pyrimidine region of the purine ring while the imidazole ring remains connected, resulting in the neutral loss of HNCO (Figure 7, Figure S3). However, it is not only the rDA reaction that leads to this neutral loss. For xanthine, QCEIMS predicted multiple pathways to yield HNCO from a variety of fragmentation pathways, including opening up the imidazole ring before the release of HNCO (Figure 7). These alternative pathways give rise to losses of HNCO and fragment ions *m/z* 109, 110, 123, 125, 137, for mass spectra X1, X2, X3, X4, and X5, respectively (Figure S3). Examples of these different types of fragmentation pathways are given for xanthine (Figure 7). This detailed investigation of trajectories and comparison of the resulting QCEIMS spectrum to the experimentally observed spectrum also gave insights into which specific ions were incorrectly predicted. For example, ions *m/z* 72 and *m/z* 80 resulted multiple times from a two-step reaction in which the opening of both rings without neutral loss occurred first, followed by fragmentation. These trajectories were not verified by the experiments. However, the direct rDA leading to *m/z* 109 as well as trajectories to *m/z* 54 and *m/z* 81 were consistent with experimental observations (Figure 7). This example showed the benefit of detailed comparisons that may be useful to improve QCEIMS.

The loss of HNCO was also the primary reaction for most lumazine derivatives (Figure S4 for the lumazine trajectory, Figure S3 for mass spectra L1–L8). Similar to xanthine trajectories, lumazine derivatives tended to undergo multiple dissociation pathways to yield HNCO neutral losses rather than only the rDA reaction. Some of their MD simulations showed a carbon monoxide loss from the pyrimidine ring of lumazine before the loss of HNCO while the pyrazine ring was connected. Some trajectories showed the loss of HNCO and CO in reverse order, and additional trajectories showed simultaneous loss of both fragments while the pyrazine ring remained intact, or losses of both HNCO and CO after both rings were broken. For benzouracil, the neutral loss of HNCO occurred after an initial loss of carbon monoxide (CO, 28 u) and not through an rDA fragmentation reaction (Figure S4).

#### 2.4.3. Loss of Hydrogen Cyanide (HCN, 27 u)

A dominant fragmentation process predicted and experimentally observed for purines and pyrimidines is the loss of hydrogen cyanide (HCN, 27 u). This reaction occurred in 89% of the tested purines and in 57% of the pyrimidines. For example, the HCN loss has been studied before for adenine [30] and was reproduced by our QCEIMS calculations. The only molecular classes that did not undergo a HCN neutral loss were pyridopyrimidines and aza-purine derivatives. For the aza-purine derivatives, the carbon atom that would have formed the HCN was substituted with a nitrogen atom, preventing HCN formation from taking place. Yet, HCN was also the most observed neutral loss for the class of hypoxanthines. For 7-methyl- and 3-methyl-hypoxanthine (spectra HX1 and HX2, Figure S3), the majority of trajectories leading to HCN losses occurred via C4–N9 and N7–C8 bond cleavages. These cleavages caused the imidazole ring to open while leaving the pyrimidine ring intact, producing fragment ions at 123 *m/z* for both methyl-hypoxanthines. When compared to the experimental spectra, only the *m/z* 123 peak of HX2 was correctly predicted, but interestingly, this peak was not present in the experimental spectra of HX1 (Figure S3). Instead, the experimental spectra of HX1 showed a peak at 96 *m/z* (M-54), which was absent in the QCEIMS predicted spectrum but might have originated from two subsequent losses of HCN. Again, such detailed investigation of differences between predicted and experimental spectra may improve future versions of the QCEIMS.

#### 2.4.4. Retro Diels–Alder Reaction of Guanines and Loss of Cyanamide (CN_2_H_2_, 42 u)

**Figure 8:**
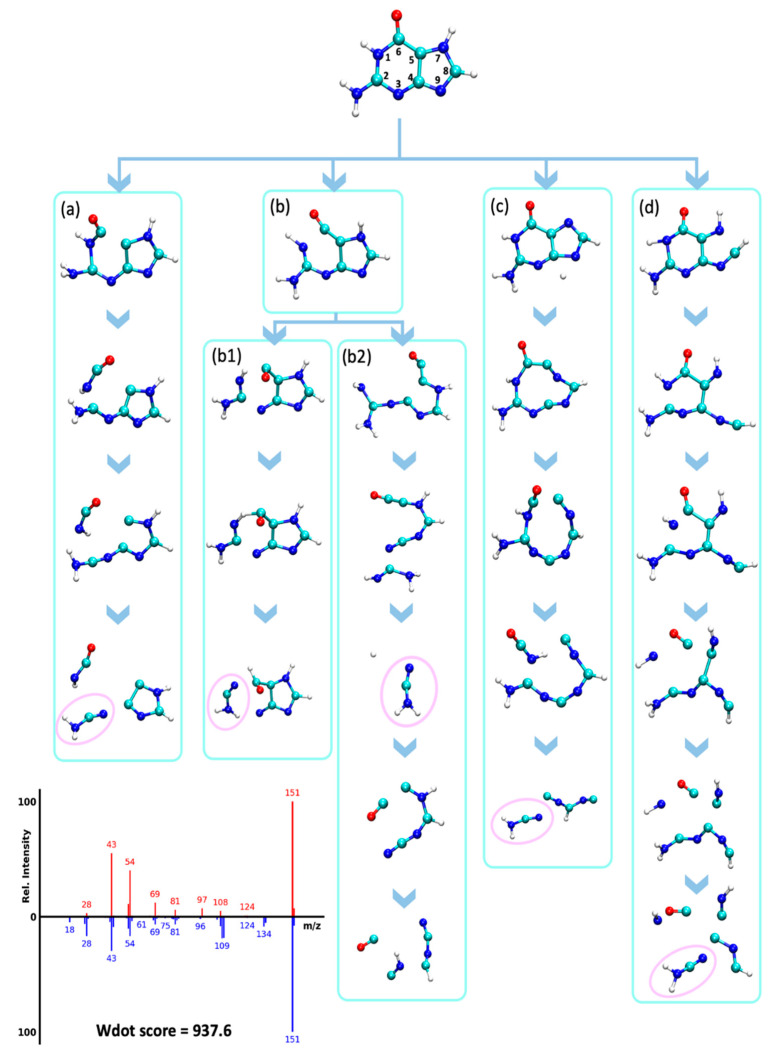
Analysis of QCEIMS trajectories for guanine marking multiple dissociation pathways (**a**–**d**) to lose cyanamide (CN_2_H_2_, 42 u, noted as pink circles). Note: none of the trajectories predicted the direct neutral loss of 42 u from the molecular ion to yield *m/z* 109. Mass spectrum insert: Top: QCEIMS, bottom: experimental spectrum.

The neutral loss of cyanamide (CN_2_H_2_, 42 u) from molecular ions has been observed experimentally in guanines and adenines [42], resulting in fragments *m/z* 109 for guanine and *m/z* 123 for methylated guanines. This neutral loss was hypothesized to occur via an rDA reaction [42]. We confirmed this hypothesis via QCEIMS trajectories that correctly predicted the presence of these neutral losses for several guanines (spectra G1-G6, Figure S3), although the abundance of these fragment ions was significantly underestimated. For instance, the guanine QCEIMS prediction yielded seven trajectories that resulted in the loss of CN_2_H_2_, corresponding to five different dissociation pathways (Figure 8). Two pathways were observed multiple times in the simulations. The first pathway was the loss of HNCO by cleaving N1-C2 and C5-C6 bonds, which was followed by a N3-C4 bond cleavage that resulted in a CN_2_H_2_ loss (Figure 8a–d). The imidazole ring was not affected by this reaction. The pathway in Figure 8b proceeded by breaking N3-C2 and C4-C5 bonds to open both rings. This reaction was followed by cleavages that resulted in either a neutral loss of CN followed by a loss of NCOH (Figure 8(b1)) or a direct neutral loss of NCOH (Figure 8(b2)). Several fragments predicted by QCEIMS were not experimentally observed, for example, *m/z* 29 for [C=OH]^+^ in Figure 8(b2,d) possibly because such ions would have too high potential energy. Destabilizing such unreasonable fragments might be a good way to improve QCEIMS in future developments.

## 3. Discussion

Quantum chemistry predictions for electron ionization mass spectra (QCEIMS) have only been performed for the past eight years [28]. We here show that QCEIMS predictions overall yielded reasonable or even excellent match scores to experimental spectra. Nevertheless, we also found a number of molecules with poor QCEIMS accuracy. Our detailed investigations of MD trajectories showed several examples such as HNCO, NCOH, and CN_2_H_2_ for which neutral losses were correctly predicted. However, we also found examples involving CN_2_H_2_ for which neutral losses were greatly underpredicted in frequency and that even yielded chemically and energetically very unlikely fragmentation structures. If QCEIMS spectral libraries are constructed to be used in identification of unknown compounds in GC-MS experimental data, it is important to understand which molecules yielded the highest likelihood of giving correct QCEIMS predictions and which molecules might have lower prediction accuracies.

With such a priori information one could build confidence models for quantum chemistry based on in silico generation of mass spectra. Unfortunately, neither our overall similarity scores of classes of purines and pyrimidines nor our detailed investigations of QCEIMS trajectories yielded such simple heuristic rules. However, other parameters were not explored, such as the rigidness of the starting molecules or the predicted fragments, or the number of rotatable bonds. When comparing QCEIMS predicted versus experimental spectra, the mass spectral similarity scores indicated significant variation across different molecules within the same structure subclasses. Yet, analyses of QCEIMS trajectories for different molecules within each subclass were surprisingly consistent. While we found mass spectral similarities to be impacted by the prediction of molecular ion abundances, QCEIMS simulations appeared to be unable to reproduce the large variations within a subgroup that were observed in experiments. We also examined other possible classifications, such as the number of rotational bonds, the number of isomers within the NIST 17 database, and the types of attached chemical groups, yet we did not find clear associations of such classifications with the accuracy of QCEIMS predictions.

One of the parameters that could be explored further is the tautomeric structure of the pyrimidines and purines as an input structure for QCEIMS models. QCEIMS methods can distinguish tautomers that consequently produce different spectra as evidenced by cytosine and guanine tautomers [27]. Calculated spectra may significantly depend on the tautomeric input structure, particularly for bonds that are involved in prototrophic shifts [27] (intramolecular proton transfers). While we did not explicitly investigate tautomers here, we found that lumazines showed significant fragmentations of neutral water losses from molecular ions that could be easily explained by tautomeric structures with hydroxyl groups. Such tautomeric hydroxyl groups were not part of our input structures; hence, the observed neutral water losses are indirect evidence supporting the need to systematically include tautomers. This large effect of tautomers on the performance of QCEIMS predictions might explain the strange bimodal distribution of weighted dot product similarity scores that we found for a range of compound classes, distinguishing well-performing from poorly performing compounds within each structure class. Future studies could investigate whether tautomers of the poorly performing structures could yield better MS similarity scores. However, exploring all possible isomers was computationally too expensive to be conducted here. An alternative reason for bimodal distributions of weighted dot product scores in molecular structure classes could be that when QCEIMS does not accurately predict the abundance of the molecular ion, it is heavily penalized by the mass-weighted dot product score, more than when using the cosine similarity score. Thus, investigating the molecular ion prediction of QCEIMS was necessary. While we found for both pyrimidines and lumazines that the incorrect prediction of the abundance of molecular ions (ranging from 98% overestimation to 79% underestimation) significantly contributed to the lowered weighted dot product scores, other factors such as tautomerism undoubtedly contributed as well.

Another possible source of error is the semiempirical model used for simulating the fragmentation trajectories, although OM2/OM3 is among the most accurate choices for semiempirical models. It is possible that running some fragmentation trajectories using ab initio methods such as hybrid density functional theory (DFT) methods, for example, could result in more accurate mass spectra, but we have not evaluated this possibility. Such studies could inform future efforts to improve the accuracy of theoretically predicted EIMS spectra.

Low dot product score mass spectral similarities occurred as a result of an inaccurate prediction of ion abundances, an overprediction of fragmentation reactions (thus creating false ions), and/or lack thereof (absent ions).

An overprediction of fragmentation reactions was not the main reason for the low score because the number of falsely predicted new ions (cut at 2% abundance) was low with an average of three ions per spectrum (see Figure S5 for more detail). Instead, low dot product scores were caused by an insufficient prediction of ions to be generated. While the overall fraction of the correctly predicted presence of fragment ions in each spectrum was reasonable with a 65% average, an underestimation of fragmentation ions also led to lower ion intensities and thus, lower dot product score matches. Overall inaccurate ratios of ion abundances may also be caused by underestimating the stability of fragment ions, leading them to undergo further fragmentation in QCEIMS.

Both purines and pyrimidines are known to undergo retro Diels–Alder reactions. Compared to purines, mass spectra of pyrimidines contained fewer fragment ions with moderate to high relative intensities. QCEIMS predictions reproduced these spectra very well, especially for the primary fragment dissociation reactions that led to abundant fragment ions. Consequently, QCEIMS predictions of pyrimidine spectra yielded better MS similarity scores than for any other class of molecules investigated here. Most likely, this performance is due to their simpler structures with only one aromatic ring. Conversely, purines have more degrees of freedom in possible fragmentation pathways because they are composed of two ring structures. Purines fragmented in more complex patterns, yielding experimental mass spectra with many more ions in varied intensities in addition to clusters of fragment ions, possibly because of hydrogen-rearrangements and use of a wide number of low frequency fragmentation pathways. However, QCEIMS predicted extra peaks that were not observed in multiple experimental mass spectra of purine-related compounds, demonstrating the difficulty of correctly following the high number of trajectories in QCEIMS. Nevertheless, QCEIMS was able to predict many of these complex fragmentation patterns in purines. Overall, we observed better QCEIMS performance for smaller and simpler structures. However, within each class of compounds, a high variance of QCEIMS performance was noted. Outliers with very low scores were not only found for purines but also for pyrimidines.

Despite the high variance of MS similarity scores of QCEIMS predicted spectra and outliers, 59 out of 80 molecules were ranked within the top 10 hits and more than half were correct in the top 5 hits when the QCEIMS predicted spectra were used to identify the molecules searching against the NIST 17 library (Table 1). Moreover, 45% of the molecules were correctly identified by matching their calculated spectra to NIST 17 library spectra as the top choice (Table 1). These data confirmed results previously reported for smaller molecules [33]. However, compounds used in our report were larger and more complex. We also showed that the use of QCEIMS predicted spectra for compound identification was directly related to the number of structurally similar molecules in the NIST 17 library. In such cases, we advise using secondary lines of evidence to further rank the most likely structure candidates, such as retention time or biological likelihood [43,44]. Correspondingly, if hits are constrained to very small libraries, false discovery rates may appear very good, while false negative hits would increase. Hence, the discovery of unexpected molecules predicted by methods like QCEIMS would be limited if based on biology- or literature-based constraints. Calculating QCEIMS spectra of novel compounds, possibly even for novel molecular classes, could become feasible in the identification of an unknown compounds in non-targeted analyses.

Besides prediction accuracy, the computational cost of QCEIMS is a crucial concern for practical applications. On average, QCEIMS took 41.6 h to calculate one mass spectrum for a system with fewer than 23 atoms. For different chemical classes, computing times varied from 30.8 h for a smaller xanthine to 63.5 h for a larger quinazolinedione (Figure S1). Computational costs are directly related to the dissociation steps in MD for which the software propagates the number of simulations by 25*x* the number of atoms in the system. However, most calculations could be performed in parallel except for the starting ground-state MD. While in silico spectra can be computed on personal workstations, the use of computer clusters to parallelize the computations on multiple nodes is advantageous, especially if predicting spectra of large numbers of molecules (thousands or more) is desired.

## 4. Methods

### 4.1. Selection of the Sample Molecules and Preparation of Their 3D Structure

To test the performance of QCEIMS predictions, we selected experimental 70 eV mass spectra of 80 different underivatized purines and pyrimidines from the NIST 17 database. The molecules were selected via a Chem Axon substructure search. We generated three-dimensional molecular structures of all 80 compounds with Avogadro2 [45] and then performed a coarse minimization for geometry optimization using the Universal Force Field (UFF). The molecular structures were converted to Mol (*.mol) and TurboMol (*.tmol) coordinate files for input into the QCEIMS software using OpenBabel (v2.3.90) [46].

### 4.2. QCEIMS Calculation

We used the 2013 release for the QCEIMS algorithm to produce all in silico electron ionization (EI) MS spectra [23]. QCEIMS calls other software programs as part of its overall procedure of EI-MS prediction. First, QCEIMS used the semiempirical program MNDO99 [47] with the orthogonalization-corrected method OM2 [48] to perform energy minimization of the input structure, followed by a ground state MD simulation that is initially equilibrated to 500 ± 50 K in the constant temperature (NVT) ensemble, followed by 50 ps of energy-conserving (NVE) dynamics using the velocity Verlet algorithm. From the NVE trajectory, snapshots at regular intervals of 1 ps were selected as the initial positions and velocities for fragmentation simulations. In order to approximate the internal excess energy (IEE) resulting from electron scattering with a typical electron kinetic energy of 70 eV, the neutral molecule was ionized by removing one electron from the HOMO, then the initial velocities were scaled such that the total kinetic energy matched a sample taken from a Poisson distribution with an average value of 0.6 eV per atom [23]. This process often led to fragmentation of the molecular ion. After the velocity scaling, a number of production runs equal to 25× the number of atoms in the molecular ion were simulated in the NVE ensemble with 0.5 fs time steps. The QCEIMS program analyzed each simulation trajectory to identify fragmentation events. Following fragmentation, charges were assigned to ions by estimating the ionization potential of each fragment at the PBE0-D3/SV(p) level of theory followed by Boltzmann weighting; these DFT calculations used the Orca software (version 3.0.0) [49]. Calculations were submitted via a Slurm workload manager to a compute cluster consisting of up to three Intel Xeon E5-2669A (2.40 GHz, 88 threads) nodes with 128 gigabytes of RAM, using 64 cores and 8 GB per QCEIMS calculation.

### 4.3. Analysis and Evaluation of Mass Spectra

Trajectories from the QCEIMS calculations were investigated via the Visual Molecular Dynamics software (VMD) [50]. Mass spectra were matched to experimental EI spectra by calculating the weighted dot product and cosine similarity scores [15], see Equations (1)–(3):

(1) $$Cos=\frac{\;\Sigma I_{L}I_{U}}{\sqrt{\Sigma I_{L}^{2}\Sigma I_{U}^{2}}}\times A,$$

(2) $$Weighted\;Dot=\frac{\;\Sigma W_{L}W_{U}}{\sqrt{\Sigma W_{L}^{2}\Sigma W_{U}^{2}}}\times A,$$

(3) $$W=[Peak\;Intensity{]}^{0.6}[Mass{]}^{3}.$$

Here, *I* refers to un-weighted intensities, and *W* refers to weighted intensities calculated according to Equation (3). The subscript *U* refers to the calculated values, the subscript *L* refers to the empirical data, and the summation is over the union of all computed (*U*) and experimental (*L*) peaks. The scaling factor *A* is 1000. The weights *m* and *n* for calculating *W* were chosen as 0.6 and 3.0 [15].

## 5. Conclusions

QCEIMS calculations work sufficiently well to be used for small molecule fragmentation predictions of aromatic and ring-structure compounds. Similarity scores of >600 were routinely achieved, and few erroneous fragment ions were predicted. Further improvements in QCEIMS software may include a more systematic approach to the potential energy surface. In addition, tautomeric structures need to be comprehensively predicted as input into molecular ensembles. Computational times averaged at approximately 41 h per compound using a 44 core CPU node for molecules with fewer than 23 atoms, meaning that QCEIMS can be deployed for large arrays of computed molecular structures if cloud computing resources are used.

## Figures and Tables

**Table 1:** Ranking the number of correct structure identifications when matching 80 QCEIMS purine and pyrimidine spectra molecules against the full NIST 17 library.

	Top 1	Top 2	Top 3	Top 4	Top 5	Top 10	Top 100	Out of Range	Total
Cumulative Number of Molecules	36	45	46	49	52	59	69	11	80
Cumulative Percentage	45%	56%	58%	61%	65%	74%	86%	14%	100%
Cumulative Wdot Average	713	711	702	702	701	712	708	326	656

**Table 2:** Differences in the relative abundances of the molecular ions (as percent of the base peak in each mass spectrum) comparing 80 QCEIMS spectra in the 12 subclasses of purines and pyrimidines to molecular ions of NIST 17 experimental spectra.

	Mol ID	Molecular Weight	Experimental Abundance (%)	QCEIMS Abundance (%)	Abundance Difference (%)	Average Difference (%)
**Pyrimidines**	5	126	82	32	−50	30.3%
	16	140	64	98	+34	
	17	140	100	71	−29	
	19	141	100	91	−9	
	41	154	100	65	−35	
	42	154	80	57	−23	
	43	155	100	97	−3	
	81	170	100	41	−59	
**Xanthines**	39	153	94	100	+6	39.8%
	75	168	27	100	+73	
	100	180	100	57	−43	
	106	181	25	67	+42	
	132	195	14	49	+35	
**Hypoxanthines**	28	150	100	83	−17	30%
	31	150	70	100	+30	
	52	164	100	57	−43	
**Lumazines**	65	166	2	100	+98	42%
	96	180	100	64	−36	
	121	192	100	79	−21	
	128	194	100	87	−13	
**Quinazoline-** **diones**	86	177	60	26	−34	19.7%
	115	190	100	87	−13	
	120	192	100	88	−12	
**Remycins**	92	179	66	19	−47	37.5%
	105	181	1	49	+48	
	123	193	24	40	+16	
	125	193	63	27	−36	
	126	193	100	31	−69	
	147	207	14	23	+9	
**Pyridopyrimidine-(1H,3H)-diones**	47	163	100	82	−18	20.4%
	50	163	88	100	+12	
	116	191	100	84	−16	
	117	191	100	54	−46	
	118	191	100	90	−10	
**Others**	70	167	11	89	+78	62.3%
	74	168	100	59	−41	
	80	170	100	21	−79	
	122	192	49	100	−51	

## Data Availability

QCEIMS mass spectra are freely available at MassBank of North America, https://massbank.us, accessed on 11 December 2021.

## Supplementary Materials

The following supporting information can be downloaded at: https://www.mdpi.com/article/10.3390/metabo12010068/s1. Figure S1: Computational costs for QCEIMS prediction of 80 purines and pyrimidines; Figure S2: The ability of QCEIMS to predict major fragment ions with abundance >20% of the base peak ion, distinguished by 12 subclasses of purines and pyrimidines; Figure S3: 32 examples of QCEIMS predicted mass spectra (top) versus NIST17 experimental mass spectra (bottom) for purines and pyrimidines. Figure S4: Analysis of QCEIMS trajectories marking the neutral loss of isocyanic acid (43 u, pink circle). (a) Benzouracil. (b) Lumazine; Figure S5: List of molecular structures with the numbers of missing, matching, and extra ions generated by QCEIMS compared to NIST17 experimental mass spectra; Table S1: Exact masses, formulas, number of fragment ions (‘peaks’) in QCEIMS mass spectra and comparison to NIST17 targets with Wdot and cosine scores, and the matching rank when querying the overall NIST17 library.
