# Supplementary material for: Evaluating the Accuracy of the QCEIMS Approach for Computational Prediction of Electron Ionization Mass Spectra of Purines and Pyrimidines
Source: Metabolites. 2022 Jan 12;12(1):68. doi: 10.3390/metabo12010068 (PMC8779335; doi:10.3390/metabo12010068)
Supplement: Supplementary file 1 [file metabolites-12-00068-s001.zip › metabolites-1528116-SM publication.pdf]

Supplements to

Evaluating the accuracy of the QCEIMS approach for computational prediction of electron ionization mass spectra of purines and pyrimidines

Jesi Lee[1,2], Tobias Kind[2], Dean Tantillo[1], Lee-Ping Wang[1], Oliver Fiehn[2*]

[1] Department of Chemistry, University of California, Davis
[2] West Coast Metabolomics Center, University of California, Davis

Supplement 1: Computational costs for QCEIMS prediction of 80 purines and pyrimidines.

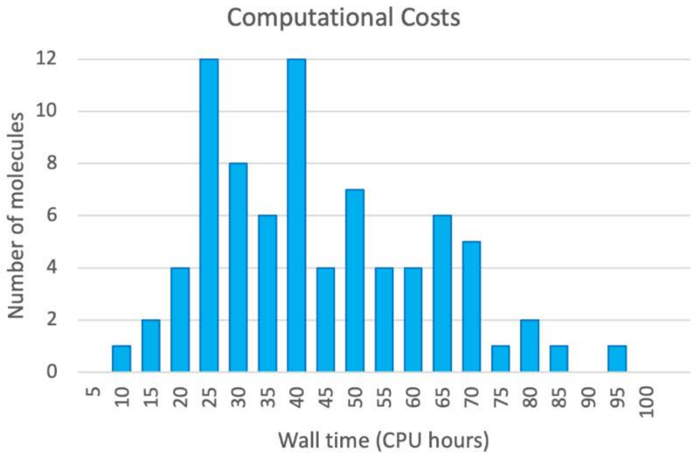

Types of molecules	Num. of mols	Total time	Avg. time
Pyrimidines	14	468.90	33.49
Methylpurines	4	183.30	45.83
Amino/hydroxy-purines	5	185.19	37.04
Adenines	5	183.51	36.70
Xanthines	8	246.43	30.80
Hypoxanthines	3	94.64	31.55
Guanines	6	264.43	44.07
Lumazines	8	339.48	42.44
Quinazdine-diones	7	444.57	63.51
Reumycins	7	298.52	42.65
Pyridopyrimidines	13	583.61	54.02
others	7	259.47	37.07
		3292.59	41.16

Supplement 2: Exact masses, formulas, number of fragment ions ('peaks') in QCEIMS mass spectra and comparison to NIST17 targets with Wdot and cosine scores, and the matching rank when querying the overall NIST17 library.

	Num	EXACT MASS	Formula	NAME	SYNONYMS	PEAK #'s	Wdot	cos	Rank
Group 1 Pyrimidines	2	126.042927	C5H4N2O2	Thymine	2,4(1H,3H)-Pyrimidinedione, 5-methyl-<br>Thymin<br>2,4-Dihydroxy-5-methylpyrimidine<br>5-Methyluracil<br>5-Methyl-2,4-dioxypyrimidine<br>5-Methyl-2,4(1H,3H)-pyrimidinedione	62	963.1	750.3	1
	3	126.042927	C5H4N2O2	6-Methyluracil	2,4(1H,3H)-Pyrimidinedione, 6-methyl-<br>2(1H)-Pyrimidinone, 4-hydroxy-6-methyl-<br>Pseudothymine<br>Uracil, 6-methyl-<br>2,4-Dihydroxy-6-methylpyrimidine<br>2,4-Pyrimidinediol, 6-methyl-<br>AWD 23-15<br>6-Methyl-1H-pyrimidine-2,4-dione<br>4-(6)-Methyluracil	62	978.94	916.9	1
	5	126.042927	C5H5N2O2	Uracil, 1-methyl-	1-Methyluracil<br>2,4(1H,3H)-Pyrimidinedione, 1-methyl-<br>1-Methyl-2,4(1H,3H)-pyrimidinedione #	54	925.32	586.1	7
	6	127.038177	C4H3N3O2	2,4(1H,3H)-Pyrimidinedione, 5-amino-	Uracil, 5-amino-<br>2,4-Dihydroxy-5-aminopyrimidine<br>5-Amino-2,4-dihydroxypyrimidine<br>5-Aminouracil<br>5-Amino-2,4(1H,3H)-pyrimidinedione #	51	983.56	909.6	2
	7	127.038177	C4H3N3O2	4-Amino-2,6-dihydroxypyrimidine	6-Amino-2,4-dihydroxypyrimidine<br>6-Aminouracil<br>4-Aminouracil<br>2,4(1H,3H)-Pyrimidinedione, 6-amino-<br>Cytosine, 6-hydroxy-<br>Uracil, 6-amino-<br>4-Amino-2,6-dihydroxylpyrimidine<br>4-Amino-2,6-dioxypyrimidine<br>4-Amino-2,6-pyrimidinediol	69	983.92	877.2	5
	8	130.017856	C4HFN2O2	5-Fluorouracil	2,4(1H,3H)-Pyrimidinedione, 5-fluoro-<br>Fluorouracil<br>Uracil, 5-fluoro-<br>Efudex<br>Fluoroplex<br>Fluracil<br>Fluril<br>NSC 19893<br>Ro 2-9757	67	979.41	937.6	1
	16	140.058578	C6H7N2O2	2,4(1H,3H)-Pyrimidinedione, 3,6-dimethyl-	Uracil, 3,6-dimethyl-<br>3,6-Dimethyl-2,4(1H,3H)-pyrimidinedione<br>3,6-Dimethyluracil	22	168.5	718.9	1
	17	140.058578	C6H8N2O2	2,4(1H,3H)-Pyrimidinedione, 1,3-dimethyl-	Uracil, 1,3-dimethyl-<br>N,N'-Dimethyluracil<br>N1,N3-Dimethyluracil<br>1,3-Dimethyluracil<br>2,4-Dihydroxy-1,3-dimethylpyrimidine<br>1,3-Dimethyl-2,4(1H,3H)-pyrimidinedione #	54	971.41	872.6	1
	18	140.058578	C6H6N2O2	2,4-Dihydroxy-5,6-dimethylpyrimidine	5,6-Dimethyluracil<br>2,4(1H,3H)-Pyrimidinedione, 5,6-dimethyl-<br>Uracil, 5,6-dimethyl-<br>4,5-Dimethyluracil<br>5,6-Dimethyl-2,4(1H,3H)-pyrimidinedione #	74	944.6	896.3	1
	19	141.053826	C5H6N3O2	2,4(1H,3H)-Pyrimidinedione, 6-amino-1-methyl-	6-Amino-1-methyl-1H-pyrimidine-2,4-dione<br>6-Amino-1-methyl-2,4(1H,3H)-pyrimidinedione #<br>6-Amino-1-methyluracil	45	236.98	377.1	4
	41	154.074227	C7H10N2O2	2,4(1H,3H)-Pyrimidinedione, 1,3,5-trimethyl-	Uracil, 1,3,5-trimethyl-<br>1,3-Dimethylthymine<br>1,3,5-Trimethyl-2,4(1H,3H)-pyrimidinedione #	79	939.83	744.6	1

	Num	EXACT MASS	Formula	NAME	SYNONYMS	PEAK #'s	Wdot	cos	Rank
	42	154.074227	C7H10N2O2	2,4(1H,3H)-Pyrimidinedione, 1,3,6-trimethyl-	Uracil, 1,3,6-trimethyl-<br>1,3,6-Trimethyl-2,4(1H,3H)-pyrimidinedione<br>1,3,6-Trimethyluracil	66	943.55	668.7	2
	43	155.069476	C6H9N3O2	6-Amino-1,3-dimethyluracil	4-Amino-1,3-dimethyl-2,6-dihydroxypyrimidine<br>4-Amino-1,3-dimethyluracil<br>2,4(1H,3H)-Pyrimidinedione, 6-amino-1,3-dimethyl-<br>Uracil, 6-amino-1,3-dimethyl-<br>1,3-Dimethyl-6-amino-uracil<br>6-Amino-1,3-dimethyl-2,4(1H,3H)-pyrimidinedione #	59	955.28	763.2	1
	81	170.080376	C6H10N4O2	5,6-Diamino-1,3-dimethyluracil	2,4(1H,3H)-Pyrimidinedione, 5,6-diamino-1,3-dimethyl-<br>Uracil, 5,6-diamino-1,3-dimethyl-<br>4,5-Diamino-1,3-dimethyluracil<br>5,6-Diamino-1,3-dimethyl-2,4(1H,3H)-pyrimidinedione #	127	958.09	586.5	48
Group 2<br>Methylpurines	1	120.043596	C5H3N4	9H-Purine	7H-Purine<br>1H-Purine<br>Purine<br>.beta.-Purine<br>Isopurine<br>3,5,7-Triazaindole<br>6H-Imidazo[4,5-d]pyrimidine<br>Imidazo(4,5-d)pyrimidine<br>NSC 753	21	338.64	383.2	2
	9	134.059246	C6H5N4	1H-Purine, 2-methyl-	Purine, 2-methyl-<br>2-Methyl-9H-purine #	45	957.27	936.5	2
	10	134.059246	C6H5N4	1H-Purine, 6-methyl-	Purine, 6-methyl-<br>6-Methylpurine<br>6-Methyl-1H-purine<br>6-Methyl-9H-purine #	22	120.76	174.6	5
	11	134.059246	C6H5N4	1H-Purine, 8-methyl-	Purine, 8-methyl-<br>8-Methylpurine<br>8-Methyl-7H-purine #	53	950.07	757.3	4
Group 3<br>Amino/hydroxy-purines	13	135.054495	C5H4N5	2-Aminopurine	1H-Purin-2-amine<br>Purine, 2-amino-<br>SQ 22,451<br>SQ 22451<br>9H-Purin-2-amine #	35	139.71	219.6	1
	14	136.038510	C5H3N4O	2-Purinol	9H-Purin-2-ol #	69	874.17	843.8	15
	15	136.038510	C5H3N4O	1H-Purin-6-ol	Imidazo[5,4-d]pyrimidine, 6-hydroxy-	45	956.11	959.4	5
	22	145.038845	C6H2N5	1H-Purine-6-carbonitrile	6-Cyanopurine<br>Purine-6-carbonitrile<br>Purine, 6-cyano-<br>9H-Purine-6-carbonitrile #	9	73.029	98.2	1

	Num	EXACT MASS	Formula	NAME	SYNONYMS	PEAK #'s	Wdot	cos	Rank
	69	167.044325	C5H4N5O2	2-Amino-6,8-dihydroxypurine	1H-Purine-6,8-dione, 2-amino-7,9-dihydro-<br>2-Amino-9H-purine-6,8-diol #	117	962.34	846.3	2
Group 4 Adenines	12	135.054495	C5H4N5	Adenine	GFFGJBXGBJISGV-UHFFFAOYSA-N	2	940.27	939.2	32
	25	149.070146	C6H7N5	6-Amino-3-methylpurine	FSASIHFSFGAIJM-UHFFFAOYSA-N	2	923.7	796.3	1
	26	149.070146	C6H7N5	7H-Purin-6-amine, 7-methyl-	HCGHYQLFMPXSDU-UHFFFAOYSA-N	2	820.27	560.2	2
	27	149.070146	C6H7N5	6-Amino-1-methylpurine	HPZMWTNATZPBIH-UHFFFAOYSA-N	2	932.23	907.8	4
	32	150.065394	C5H5N6	2,6-Diaminopurine	MSSXOMSJDRHRMC-UHFFFAOYSA-N	2	116.74	100.5	N/R
Group 5 Xanthines	37	152.033425	C5H3N4O2	Xanthine	1H-Purine-2,6-dione, 3,7-dihydro-<br>Isoxanthine<br>Pseudoxanthine<br>Purine-2,6(1H,3H)-dione<br>Xan<br>Xanthic oxide<br>Xanthin<br>1H-Purine-2,6-diol<br>2,6-Dioxo-1,2,3,6-tetrahydropurine	43	949	658.7	9
	39	153.028674	C4N5O2	8-Azaxanthine	1H-1,2,3-Triazolo[4,5-d]pyrimidine-5,7(4H,6H)-dione<br>v-Triazolo(4,5-d)pyrimidine-5,7-diol<br>NSC 756<br>USAF cb-26<br>Xanthazol<br>1H-v-Triazolo(4,5-d)pyrimidine-5,7(4H,6H)-dione<br>2,6-Dioxy-8-azapurine	68	967.05	687.1	1
	66	166.049075	C6H4N4O2	2,6-Dihydroxy-7-methylpurine	7-Methylxanthine<br>1H-Purine-2,6-dione, 3,7-dihydro-7-methyl-<br>Heteroxanthin<br>Heteroxanthine<br>Xanthine, 7-methyl-<br>7-Methylxanthin	31	943.54	913.7	1
	75	168.028340	C5N4O3	Uric acid	1H-Purine-2,6,8(3H)-trione, 7,9-dihydro-<br>Lithic acid<br>Purine-2,6,8(1H,3H,9H)-trione<br>1H-Purine-2,6,8-triol<br>2,6,8-Trioxopurine<br>2,6,8-Trioxypurine<br>7,9-Dihydro-1H-purine-2,6,8(3H)-trione<br>2,6,8-Trihydroxypurine<br>8-Hydroxyxanthine	139	786.88	457.4	6
	99	180.064726	C7H7N4O2	Theobromine	1H-Purine-2,6-dione, 3,7-dihydro-3,7-dimethyl-<br>Diurobromine<br>Santheose<br>SC 15090<br>Teobromin<br>Theosalvose<br>Theostene<br>Thesal<br>3,7-Dimethylxanthine	49	949.83	762.8	1

	Num	EXACT MASS	Formula	NAME	SYNONYMS	PEAK #'s	Wdot	cos	Rank
	100	180.064726	C7H7N4O 2	Theophylline	1H-Purine-2,6-dione, 3,7-dihydro-1,3-dimethyl-<br>Elixophyllin<br>Elixophylline<br>Lanophyllin<br>Liquophylline<br>Optiphyllin<br>Parkophyllin<br>Pseudotheophylline<br>Solosin	48	953.5	732.5	1
	106	181.059975	C6H6N5O 2	3H-N-Triazolo(4,5-d)pyrimidine-5,7(4H,6H)-dione, 3,6-dimethyl-	1,9-Dimethyl-8-azaxanthin<br>3,6-Dimethyl-3H-v-triazolo(4,5-d)pyrimidine-5,7(4H,6H)-dione	32	87.83	578.9	1
	132	195.075624	C7H9N5O 2	3H-v-Triazolo(4,5-d)pyrimidine-5,7(4H,6H)-dione, 3,4,6-trimethyl-	1,3,9-Trimethyl-8-azaxanthin<br>3,4,6-Trimethyl-3H-v-triazolo(4,5-d)pyrimidine	38	254.35	515.5	2
Group 6 Hypoxanthines	28	150.054161	C6H5N4O	6H-Purin-6-one, 1,7-dihydro-7-methyl-	Hypoxanthine, 7-methyl-<br>7-Methylhypoxanthine<br>7-Methyl-6-hydroxypurine<br>7-Methyl-1,7-dihydro-6H-purin-6-one #	33	104.06	132.3	N/R
	31	150.054161	C6H5N4O	6H-Purin-6-one, 3,7-dihydro-3-methyl-	Hypoxanthine, 3-methyl-<br>N3-Methylhypoxanthine<br>3-Methylhypoxanthine<br>3-Methyl-3,7-dihydro-6H-purin-6-one #	22	207.38	543.6	1
	52	164.069811	C7H8N4O	6H-Purin-6-one, 1,7-dihydro-1,7-dimethyl-	Hypoxanthine, 1,7-dimethyl-<br>1,7-Dimethylhypoxanthine<br>1,7-Dimethyl-1,7-dihydro-6H-purin-6-one #	75	891.87	870.3	1
Group 7 Guanines	35	151.049410	C5H3N5O	Guanine	6H-Purin-6-one, 2-amino-1,7-dihydro-<br>C.I. Natural White 1<br>C.I. 75170<br>Dew Pearl<br>Guanin<br>Guanine enol<br>Hypoxanthine, 2-amino-<br>Mearlmaid<br>Natural Pearl Essence	84	937.61	912.8	2
	53	165.065060	C6H6N5O	7-Methylguanine	6H-Purin-6-one, 2-amino-1,7-dihydro-7-methyl-<br>Guanine, 7-methyl-<br>N7-Methylguanine<br>2-Amino-7-methylhypoxanthine<br>2-Amino-1,7-dihydro-7-methyl-6H-purin-6-one<br>2-Amino-7-methyl-1,7-dihydro-6H-purin-6-one<br>NSC 193444	38	127.23	381.7	2
	54	165.065060	C6H6N5O	6H-Purin-6-one, 2-amino-1,7-dihydro-1-methyl-	Guanine, 1-methyl-<br>N1-Methylguanine<br>1-Methylguanine<br>2-Amino-1-methyl-1,7-dihydro-6H-purin-6-one #	30	166.66	423.5	1
	55	165.065060	C6H6N5O	2-Amino-6-hydroxy-9-methylpurine	9-Methylguanine<br>6H-Purin-6-one, 2-amino-1,9-dihydro-9-methyl-<br>2-Amino-9-methyl-1,9-dihydro-6H-purin-6-one #	100	937.71	940	1
	56	165.065060	C6H6N5O	6H-Purin-6-one, 2-amino-3,7-dihydro-3-methyl-	Guanine, 3-methyl-<br>N3-Methylguanine<br>3-Methylguanine<br>2-Amino-3-methyl-3,7-dihydro-6H-purin-6-one #	48	230.74	518.6	1

	Num	EXACT MASS	Formula	NAME	SYNONYMS	PEAK #'s	Wdot	cos	Rank
	124	193.059975	C7H5N5O2	Acetamide, N-(6,7-dihydro-6-oxo-1H-purin-2-yl)-	N-2-Acetylguanine<br>N-(6-Oxo-6,9-dihydro-1H-purin-2-yl)acetamide #<br>#?	67	708.08	348.7	N/R
Group 8 Lumazines	51	164.033425	C6H2N4O2	Lumazine	2,4(1H,3H)-Pteridinedione<br>2,4(3H,8H)-Pteridinedione<br>2,4-Dihydroxypteridine<br>2,4-Pteridinediol<br>Lumazin<br>Pteridinedione, 2,4(1h,3h)-	92	951.94	896.2	1
	65	166.049075	C6H4N4O2	7,8-Dihydro-2,4(1H,3H)-pteridinedione		75	15.729	47.38	N/R
	88	178.049075	C7H5N4O2	2,4(1H,3H)-Pteridinedione, 8-methyl-	Lumazine, 8-methyl-<br>8-Methyl-2,4(3H,8H)-pteridinedione #	47	215.83	589.2	1
	91	178.049075	C7H4N4O2	2,4(1H,3H)-Pteridinedione, 6-methyl-	Lumazine, 6-methyl-<br>6-Methyl-2,4(1H,3H)-pteridinedione #	17	156.79	313.5	27
	95	180.028340	C6HN4O3	2,4,7(1H,3H,8H)-Pteridinetrione	Isoxantholumazine<br>Lumazine, 7-hydroxy-<br>Violapterin<br>2,4,7-Trihydroxypteridine<br>7-Hydroxylumazine<br>2,4,7-Pteridinetriol	83	909.86	615.5	8
	96	180.028340	C6HN4O3	2,4,6(3H)-Pteridinetrione, 1,5-dihydro-	Lumazine, 6-hydroxy-<br>Pteridine-2,4,6-trione<br>2,4,6(1H,3H,5H)-Pteridinetrione<br>2,4,6-Pteridinetriol<br>2,4,6-Trihydroxypteridine<br>6-Hydroxylumazine<br>1,5-Dihydro-2,4,6(3H)-pteridinetrione #	23	52.832	135.5	N/R
	121	192.064726	C8H8N4O2	2,4(1H,3H)-Pteridinedione, 1,3-dimethyl-	Lumazine, 1,3-dimethyl-<br>1,3-Dimethyllumazine<br>1,3-Dimethylpteridine-2,4-dione<br>N,N'-Dimethyllumazin<br>1,3-Dimethyl-2,4(1H,3H)-pteridinedione #<br>N,N'-Dimethyllumazine	61	941.32	786.3	1
	128	194.043990	C7H3N4O3	2,4,7(1H,3H,8H)-Pteridinetrione, 6-methyl-	2,4,7-Pteridinetriol, 6-methyl-<br>2,4(1H,3H)-Pteridinedione, 7-hydroxy-6-methyl-<br>6-Methyl-2,4,7(1H,3H,8H)-quinazolinetrione #	46	818.57	533.3	N/R
	46	162.042928	C8H4N2O2	2,4(1H,3H)-Quinazolinedione	Benzouracil<br>Benzoyleneurea<br>Quinazoline-2,4-dione<br>Quinazolinedione<br>Urea, benzoylene-<br>1,2,3,4-Tetrahydroquinazoline-2,4-dione<br>2,4-Dihydroxyquinazoline<br>(1H,3H)Quinazoline dione-2,4<br>2,4-Dioxotetrahydroquinazoline	75	953.38	846.8	9
	83	176.058578	C9H7N2O2	2,4(1H,3H)-Quinazolinedione, 3-methyl-	3-Methyl-2,4(1H,3H)-quinazolinedione	69	951.15	817.6	1

	Num	EXACT MASS	Formula	NAME	SYNONYMS	PEAK #'s	Wdot	cos	Rank
Group 9 Quinazoline-diones	84	176.058578	C9H7N2O2	2,4(1H,3H)-Quinazolinedione, 1-methyl-	Glycosmicine<br>1-Methyl-2,4(1H,3H)-Quinazolinedione<br>Methyl-1 (1H,3H)quinazolinedione-2,4	41	299.94	429.5	29
	86	177.053826	C8H6N3O2	3-Amino-1H-quinazoline-2,4-dione		60	233.5	298.9	N/R
	87	177.053826	C8H5N3O2	6-Amino-1H-quinazoline-2,4-dione		35	968.56	972.5	1
	115	190.074227	C10H10N2O2	1,3-Dimethyl-2,4(1H,3H)-quinazolinedione	2,4(1H,3H)-Quinazolinedione, 1,3-dimethyl-<br>Dimethyl-1,3 (1H,3H)quinazolinedione-2,4<br>1,3-Dimethyl-1,2,3,4-tetrahydroquinazolin-2,4-dione	76	931.74	835.7	1
	120	192.053493	C9H8N2O3	2,4(1H,3H)-Quinazolinedione, 3-hydroxy-1-methyl-	3-Hydroxy-1-methyl-2,4(1H,3H)-quinazolinedione #	20	294.24	366	N/R
Group 10 Reumycins	92	179.044325	C6H4N5O2	Reumycin	Pyrimido(5,4-e)-as-triazine-5,7(6H,8H)-dione, 6-methyl-<br>Reumitsin<br>Rheumygin<br>Pyrimido(5,4-e)-as-triazine-5,7(1H,6H)-dione, 6-methyl-<br>BA 51-090492<br>6-Methylpyrimido(5,4-e)-as-triazine-5,7(1H,6H)-dione	32	534.26	451.3	30
	105	181.023589	C5HN5O3	Pyrimido[4,5-e][1,2,4]triazine-6,8(5H,7H)-dione, 3-hydroxy-		115	606.67	505.9	N/R
	123	193.059975	C7H7N5O2	5,7-Dimethylpyrimido[4,5-E]-1,2,4-triazine-6,8(5H,7H)-dione		30	920.23	954	1
	125	193.059975	C7H7N5O2	Fervenulin	Compd. 7215<br>Fervenuline<br>FH-3582-A<br>KC 1017<br>Planomycin<br>Pyrimido(5,4-e)-as-triazine-5,7(6H,8H)-dione, 6,8-dimethyl-<br>Pyrimido(5,4-e)-1,2,4-triazine-5,7(6H,8H)-dione, 6,8-dimethyl-	39	317.18	391.7	71
	126	193.059975	C7H7N5O2	Toxoflavin	Pyrimido(5,4-e)-as-triazine-5,7(1H,6H)-dione, 1,6-dimethyl-<br>Pyrimido(5,4-e)-1,2,4-triazine-5,7(1H,6H)-dione, 1,6-dimethyl-<br>Toxoflavine<br>Xanthothricin<br>Xanthotricin<br>1,6-Dimethyl-5,7-dioxo-1,5,6,7-tetrahydropyrimido(5,4-e)-as-triazine-	31	224.79	355.5	6
	133	195.075624	C7H8N5O2	Theophylline, 8-amino-	8-Aminoteofiliny<br>8-Aminotheophylline<br>1H-Purine-2,6-dione, 8-amino-3,7-dihydro-1,3-dimethyl-<br>7H-Purine-2,6(1H,3H)-dione, 8-amino-1,3-dimethyl-<br>8-Amino-1,3-dimethyl-3,7-dihydro-1H-purine-2,6-dione #	134	971.05	825.9	1

	Num	EXACT MASS	Formula	NAME	SYNONYMS	PEAK #'s	Wdot	cos	Rank
Group 11 Pyridopyrimidines	47	163.038177	C7H3N3O2	Pyrido[3,2-d]pyrimidine-2,4(1H,3H)-dione	Pyrido[3,2-d]pyrimidine-2,4-diol 2,4-Dihydroxypyrido[3,2-d]pyrimidine	24	249.32	761	1
	48	163.038177	C7H3N3O2	Pyrido[2,3-d]pyrimidine-2,4(1H,3H)-dione		18	233.69	415.4	1
	50	163.038177	C7H3N3O2	Pyrido[4,3-d]pyrimidine-2,4(1H,3H)-dione	Pyrido[4,3-d]pyrimidin-4(3H)-one, 2-hydroxy-	37	939.71	757.7	11
	116	191.069476	C9H9N3O2	Pyrido[2,3-d]pyrimidine-2,4(1H,3H)-dione, 1,3-dimethyl-	1,3-Dimethylpyrido[2,3-d]pyrimidine-2,4(1H,3H)-dione #	28	169.72	295.1	N/R
	117	191.069476	C9H9N3O2	Pyrido[3,2-d]pyrimidine-2,4(1H,3H)-dione, 1,3-dimethyl-	1,3-Dimethylpyrido[3,2-d]pyrimidine-2,4(1H,3H)-dione #	29	302.24	696	3
	118	191.069476	C9H9N3O2	Pyrido[3,4-d]pyrimidine-2,4(1H,3H)-dione, 1,3-dimethyl-	1,3-Dimethylpyrido[3,4-d]pyrimidine-2,4(1H,3H)-dione #	32	262.9	569.7	1
Group 12 Others	38	152.058578	C7H6N2O2	1H,3H,5H,6H,7H-Cyclopenta[d]pyrimidine-2,4-dione		64	935.12	842.9	N/R
	70	167.044325	C5H3N5O2	4-Methyl-3,4-dihydro-[1,2,3]triazolo[4,5-d]pyrimidine-5,7-dione		39	347.9	46.95	N/R
	74	168.017107	C6H2N2O4	1,5-Dihydrofuro[3,4-d]pyrimidine-2,4,7(3H)-trione		47	932.84	586.8	26
	80	170.007605	C4N4O4	1-Oxy-4H-[1,2,5]oxadiazolo[3,4-d]pyrimidine-5,7-dione		33	935.41	561.2	1

	Num	EXACT MASS	Formula	NAME	SYNONYMS	PEAK #'s	Wdot	cos	Rank
	93	179.069476	C8H8N3O2	1H-Pyrrolo(2,3-d)pyrimidine-2,4(3H,7H)-dione, 1,3-dimethyl-	1,3-Dimethyl-1H-pyrrolo(2,3-d)pyrimidine-2,4(3H,7H)-dione<br>1H-Pyrrolo[2,3-d]pyrimidine-2,4(3H,7H)-dione, 1,3-dimethyl-<br>1H-Pyrrolo[3,2-d]pyrimidine-5,7(4H,6H)-dione, 4,6-dimethyl-<br>1H-Pyrrolo[3,2-d]pyrimidine-2,4(3H,5H)-dione, 1,3-dimethyl-	79	885.54	488.4	1
	122	192.064726	C8H6N4O2	1H-Pyrrolo[2,1-f]purine-2,4(3H,6H)-dione, 7,8-dihydro-		95	934.63	503.5	1
	146	207.075624	C8H9N5O2	Pyrimido[5,4-E][1,2,4]triazine-5,7(1H,6H)-dione, 1,3,6-trimethyl-		86	911.02	310.8	45

Supplement 3: The ability of QCEIMS to predict major fragment ions with abundance >20% of the base peak ion, distinguished by 12 subclasses of purines and pyrimidines. *Note*: Most major fragment ions were correctly predicted by QCEIMS for most structure subclasses. While QCEIMS predicted less than 20% of additional fragment ions not verified by experimental spectra, more than 40% extra fragment ions were predicted by QCEIMS of methylpurines.

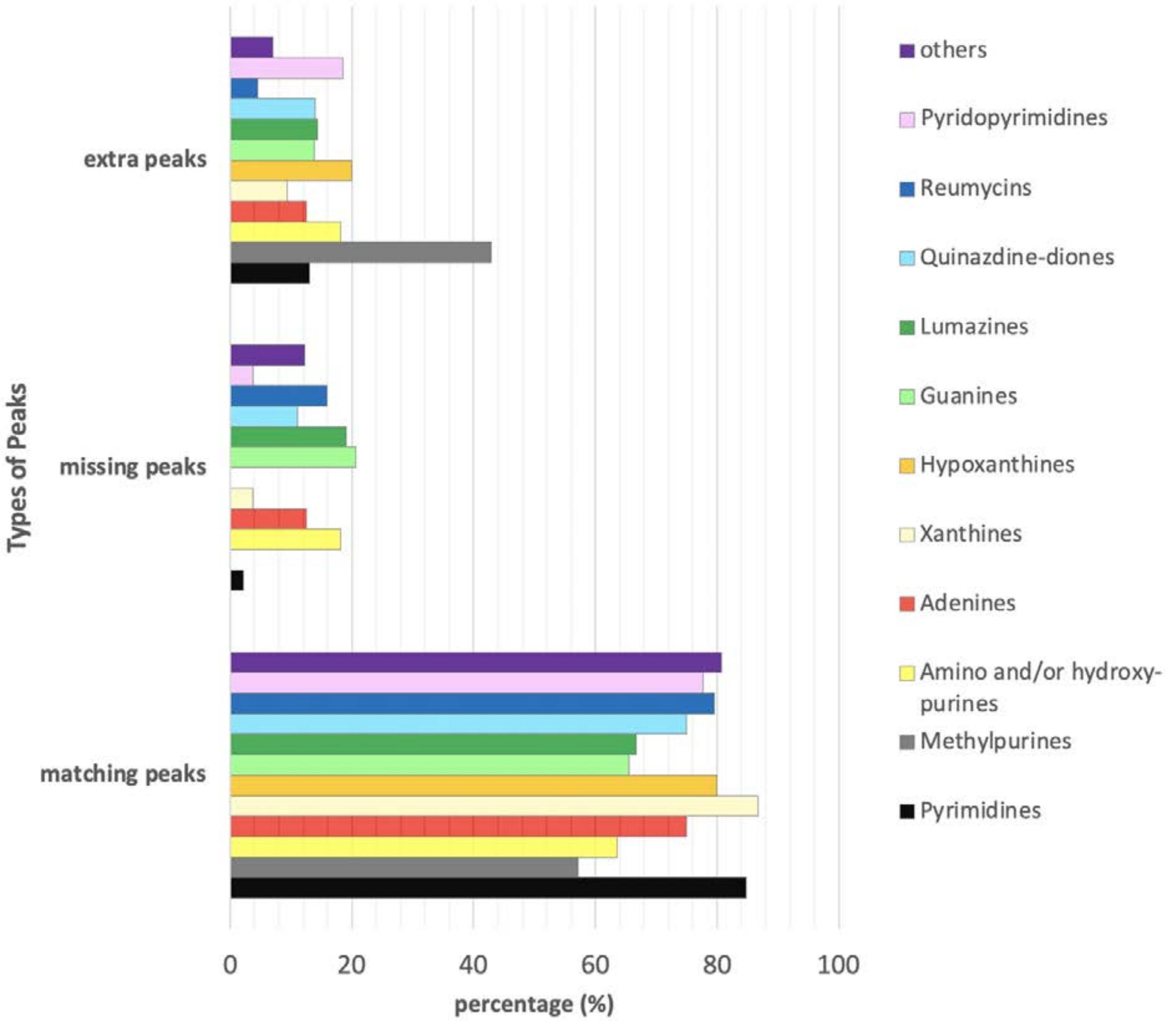

Supplement 4: 32 examples of QCEIMS predicted mass spectra (top) versus NIST17 experimental mass spectra (bottom) for purines and pyrimidines.

(P1) 6-methyluracil

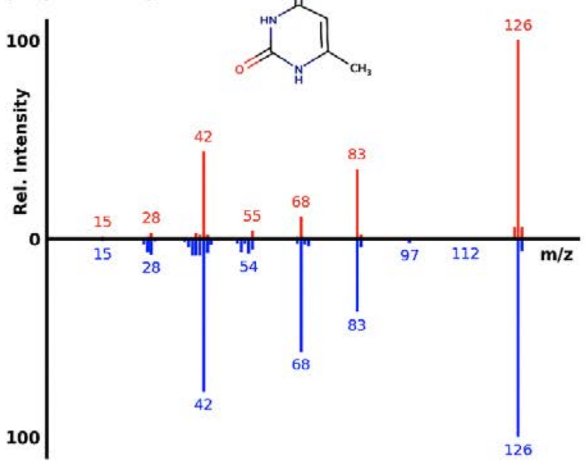

(P2) 1-methyluracil

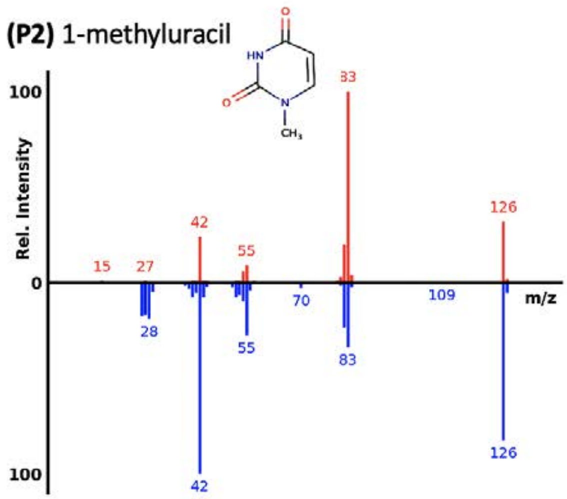

(P3) 5-aminouracil

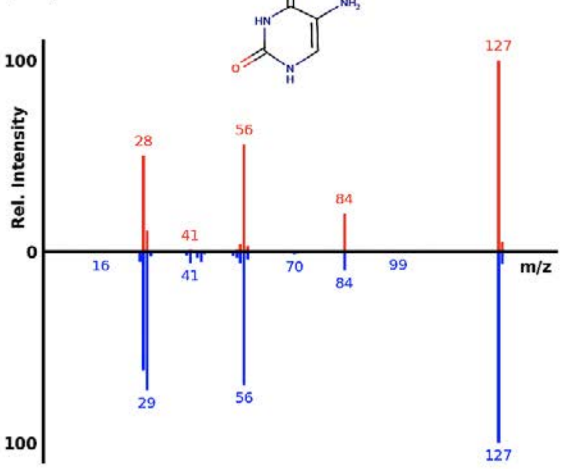

(P4) 5-fluorouracil

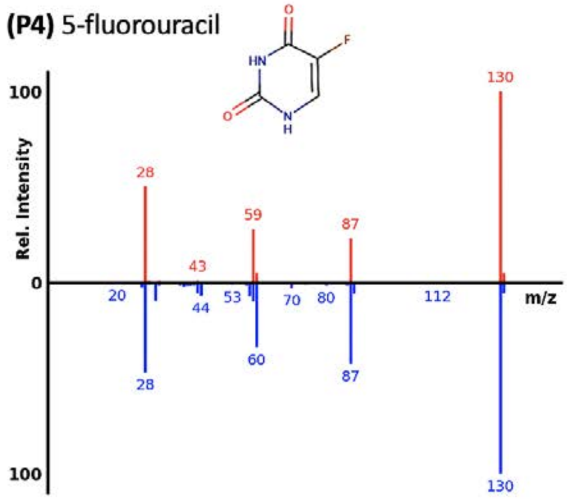

(P5) 3,6-dimethyluracil

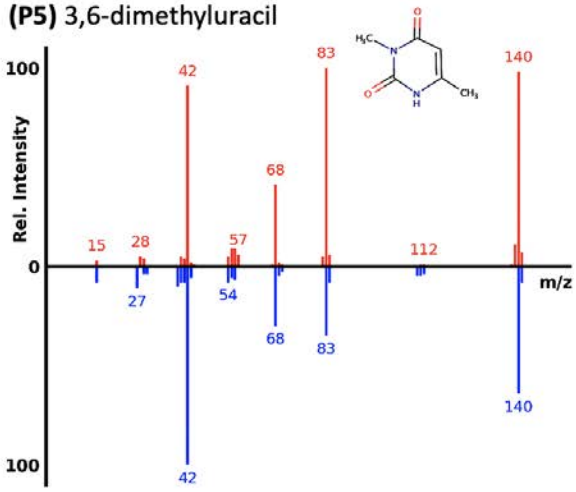

(P6) 1,3-dimethyluracil

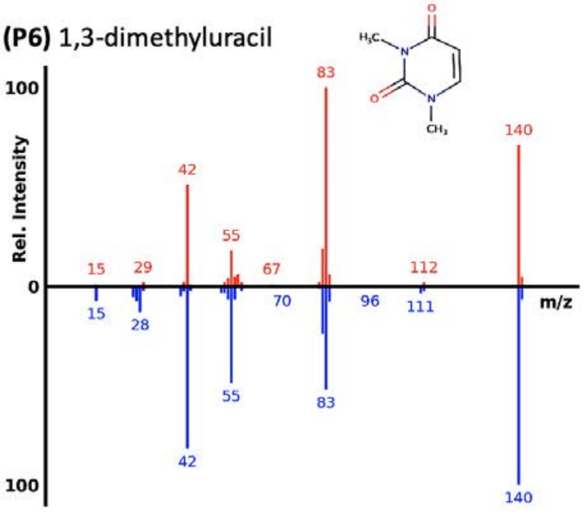

(P7) 6-amino-1-methyluracil

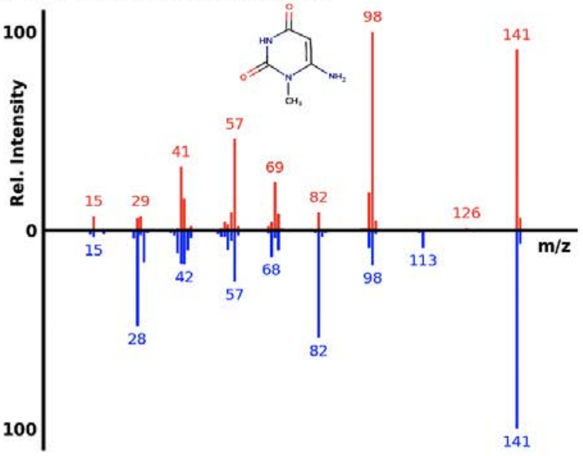

(X1) Xanthine

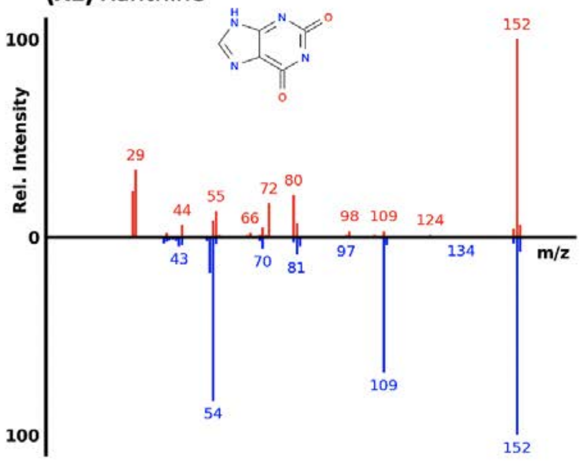

(X2) 8-azaxanthine

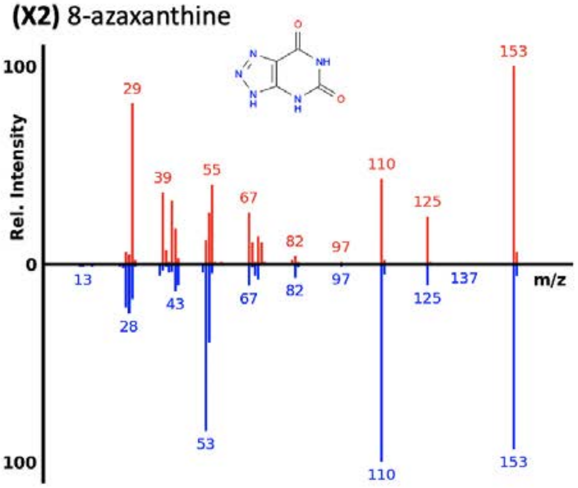

(X3) 7-methylxanthine

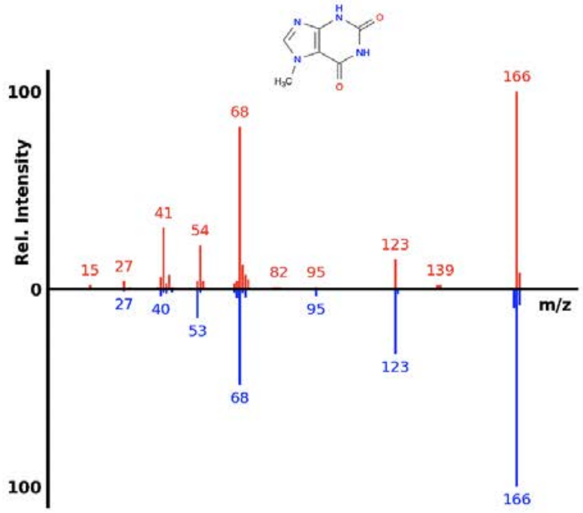

(X4) Uric acid (8-hydroxyxanthine)

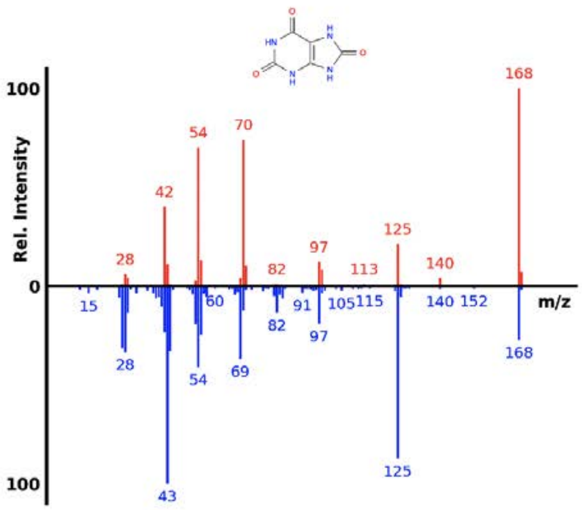

(X5) 3,7-dimethylxanthine

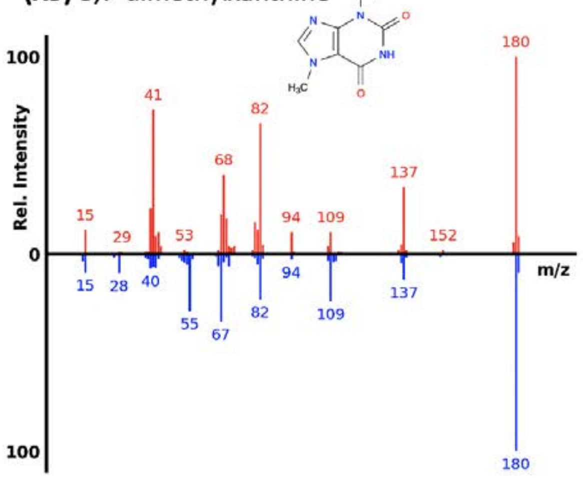

(X6) 1,3-dimethylxanthine

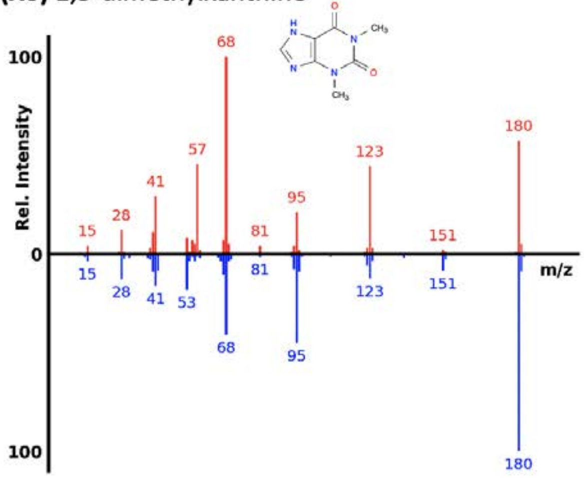

(X7) 1,9-dimethyl-8-azaxanthine

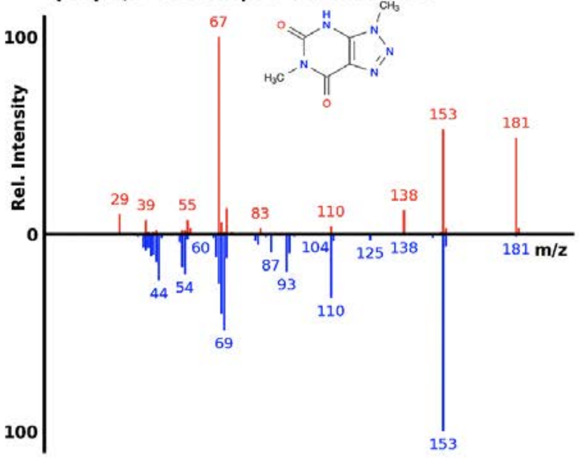

(X8) 1,3,9-trimethyl-8-azaxanthine

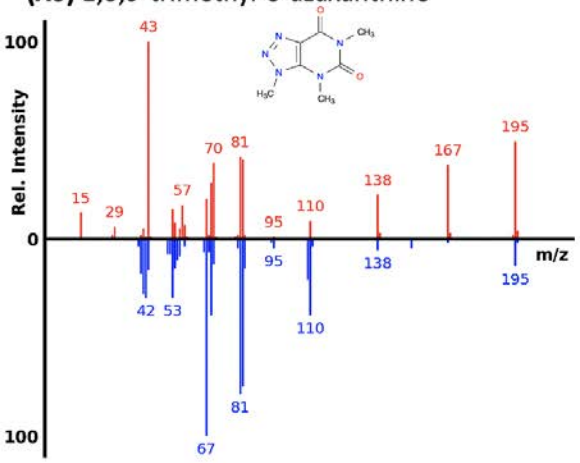

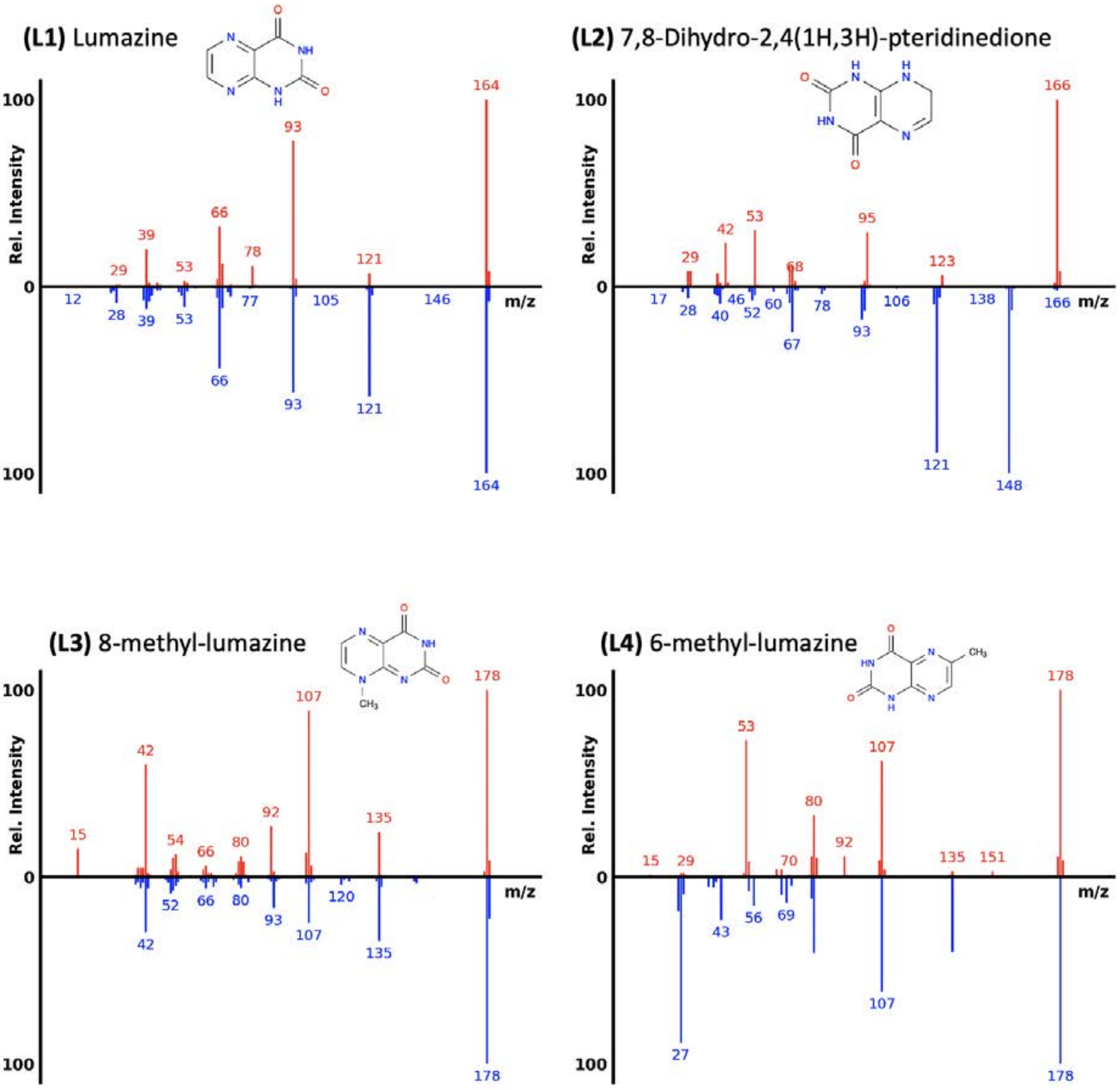
(L1) Lumazine
(L2) 7,8-Dihydro-2,4(1H,3H)-pteridinedione
(L3) 8-methyl-lumazine
(L4) 6-methyl-lumazine
Rel. Intensity
m/z
100
0
100

(L5) 7-hydroxy-lumazine

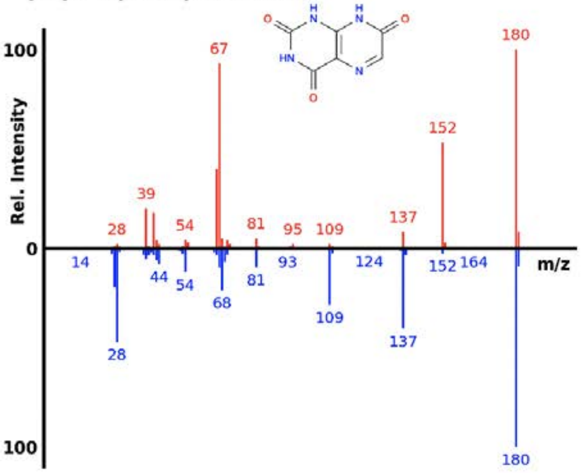

(L6) 6-hydroxy-lumazine

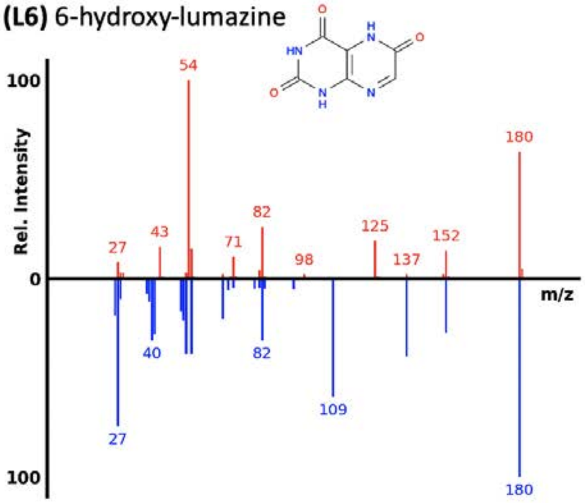

(L7) 1,3-dimethyl-lumazine

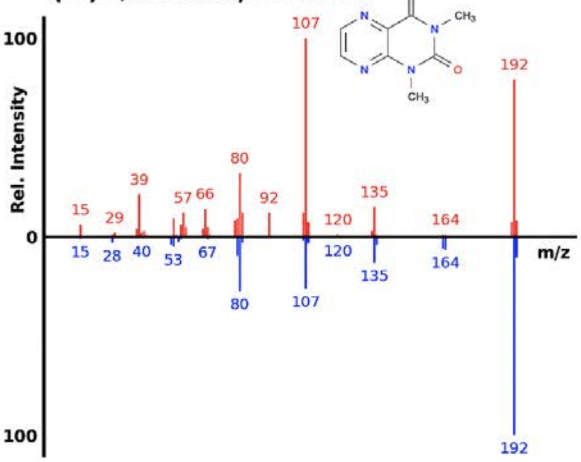

(L8) 6-methyl-2,4,7-Pteridinetriol

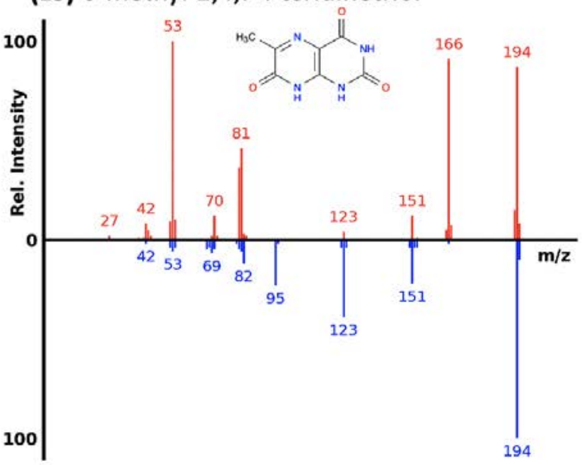

(HX1) Hypoxanthine

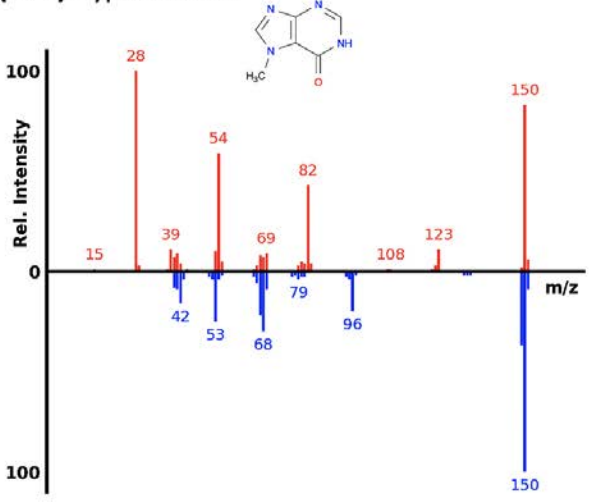

(HX2) 3-methylhypoxanthine

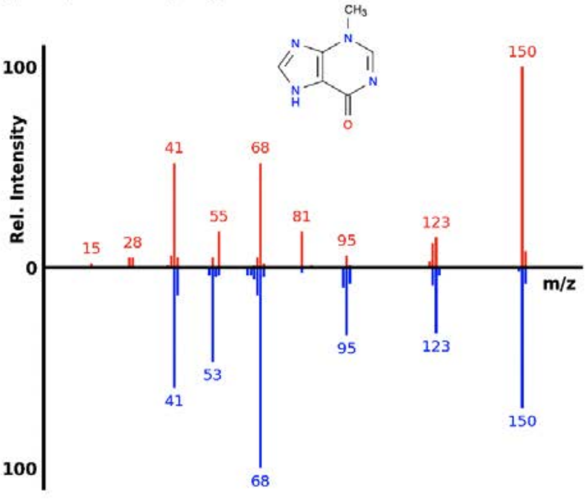

(HX3) 1,7-dimethylhypoxanthine

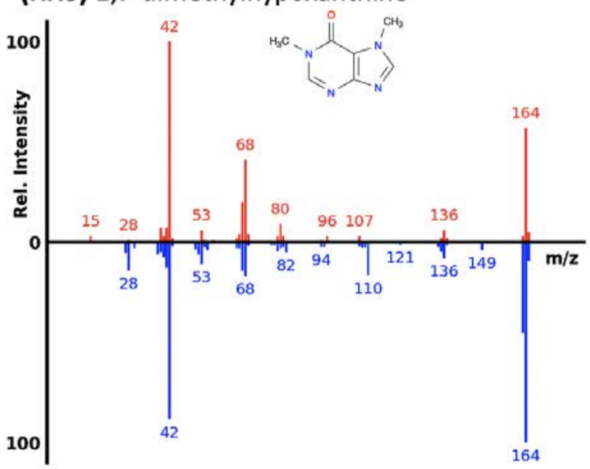

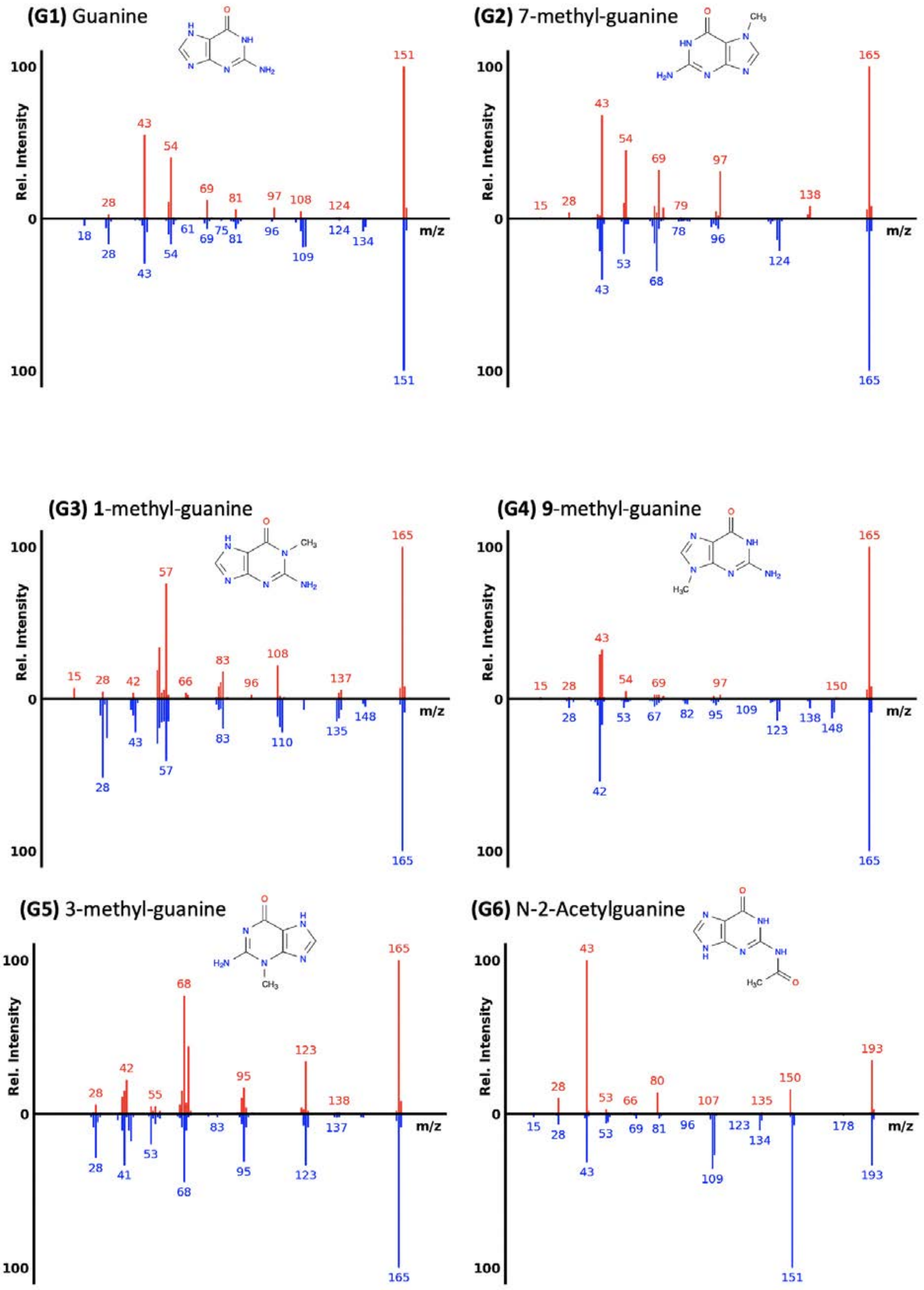
(G1) Guanine
(G2) 7-methyl-guanine
(G3) 1-methyl-guanine
(G4) 9-methyl-guanine
(G5) 3-methyl-guanine
(G6) N-2-Acetylguanine
Rel. Intensity
m/z
100
0
100
151
43
54
28
69
81
97
108
124
18
61
75
96
109
134
165
15
79
138
53
68
78
57
42
66
83
110
135
137
148
150
67
82
95
123
55
41
193
80
107
178

Supplement 5 Analysis of QCEIMS trajectories marking the neutral loss of isocyanic acid (43 u, pink circle). (a) Benzouracil. (b) Lumazine.

(a)

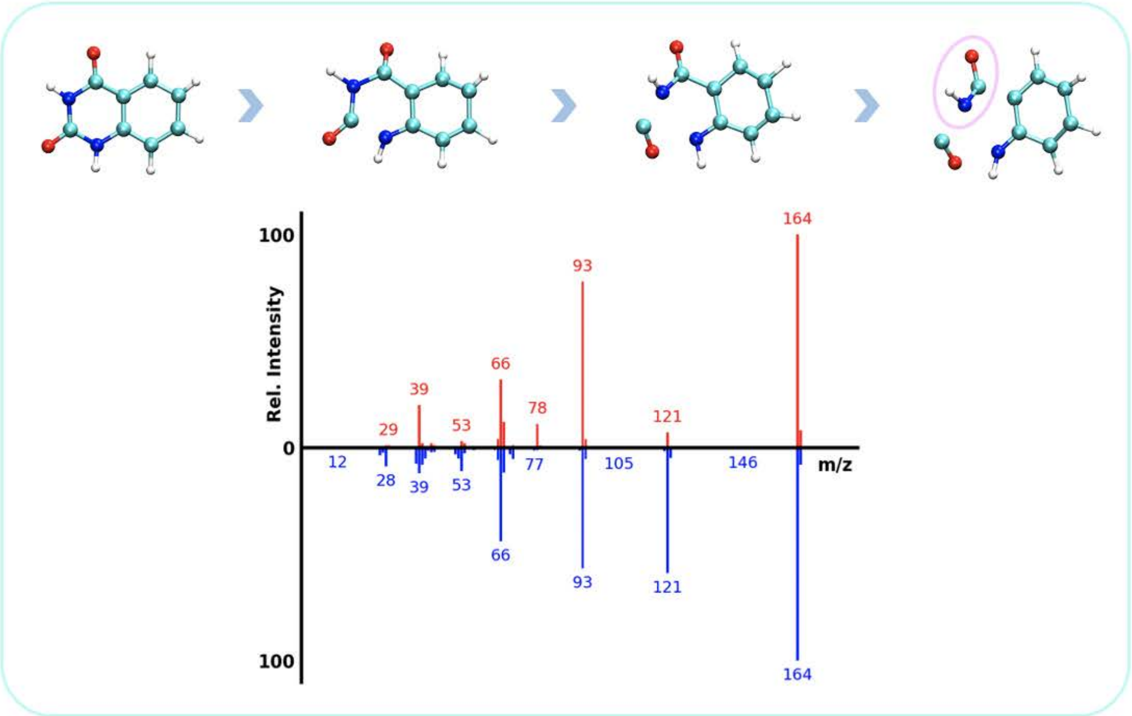

(b)

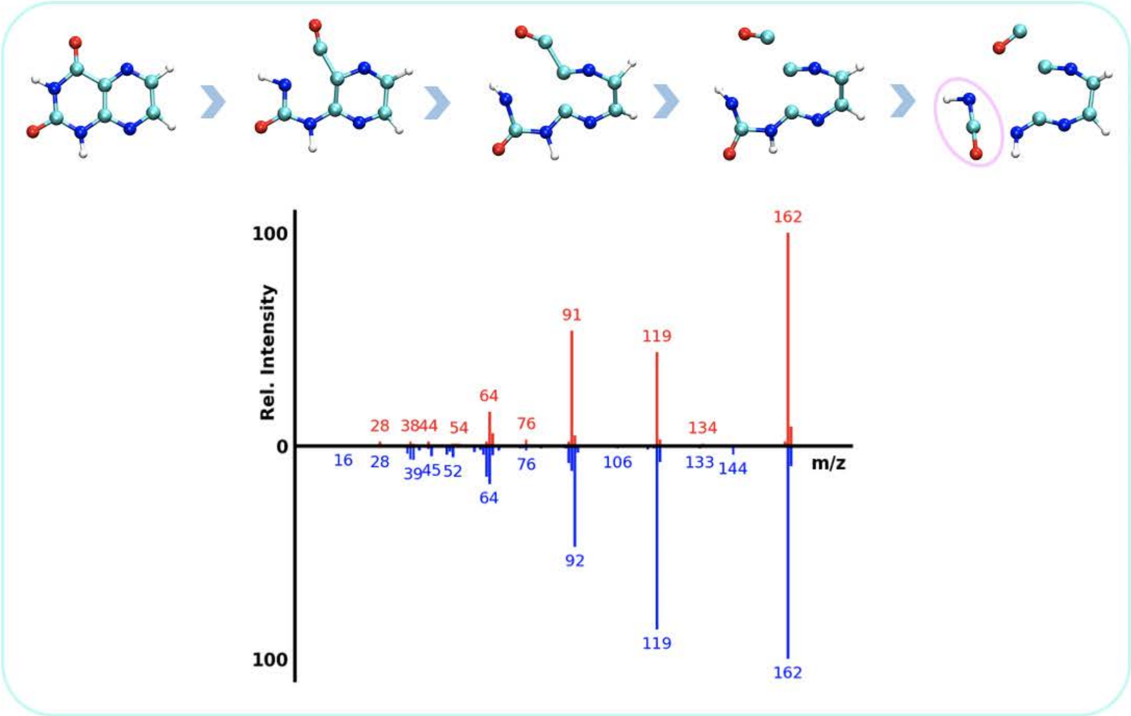

Supplement 6: List of molecular structures with the numbers of missing, matching, and extra ions generated by QCEIMS compared to NIST17 experimental mass spectra.

Group	Mol ID	Structure	# of matching peaks	# of missing peaks	# of extra peaks
Pyrimidines	2	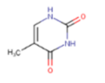	12	7	1
Pyrimidines	3	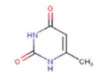	13	11	1
Pyrimidines	5	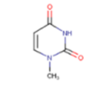	14	7	0
Pyrimidines	6	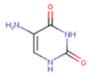	9	6	1
Pyrimidines	7	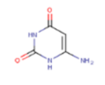	10	12	0
Pyrimidines	8	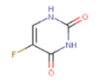	6	6	1
Pyrimidines	16	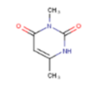	16	4	6
Pyrimidines	17	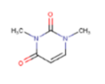	15	6	1
Pyrimidines	18	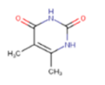	14	13	1
Pyrimidines	19	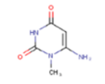	20	6	2
Pyrimidines	41	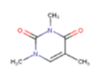	19	6	1
Pyrimidines	42	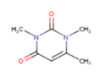	19	10	2
Pyrimidines	43	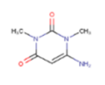	16	7	1
Pyrimidines	81	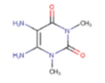	16	22	0
Methylpurines	1	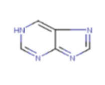	8	4	5
Methylpurines	9	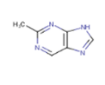	11	1	1
Methylpurines	10	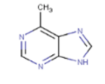	9	6	5

Group	Mol ID	Structure	# of matching peaks	# of missing peaks	# of extra peaks
Methyl-purines	11	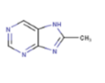	15	3	1
Amino/hydroxypurines	13	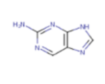	11	7	2
Amino/hydroxypurines	14	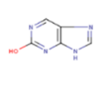	11	13	1
Amino/hydroxypurines	15	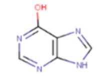	4	6	3
Amino/hydroxypurines	22	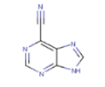	4	0	6
Amino/hydroxypurines	69	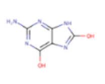	14	17	0
Adenines	12	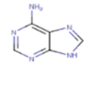	8	7	1
Adenines	25	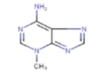	19	5	2
Adenines	26	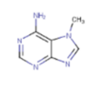	24	3	1
Adenines	27	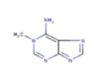	18	16	1
Adenines	32	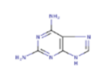	7	4	5
Xanthines	37	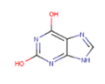	10	5	5
Xanthines	39	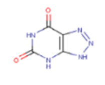	19	2	1
Xanthines	66	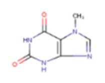	11	2	3
Xanthines	75	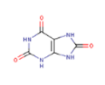	19	24	1
Xanthines	99	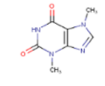	24	6	4
Xanthines	100	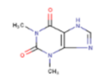	21	5	1

Class	Mol ID	Structure	# of matching peaks	# of missing peaks	# of extra peaks
Xanthines	106	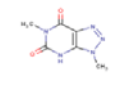	18	5	10
	132	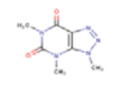	19	8	8
Hypoxanthines	28	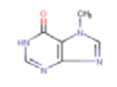	14	7	8
	31	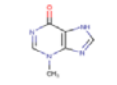	13	6	6
	52	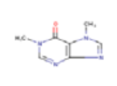	21	13	2
Guanines	35	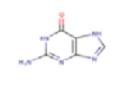	10	11	1
	53	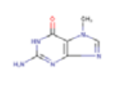	13	9	6
	54	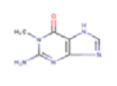	17	11	6
	55	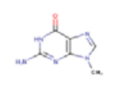	10	15	0
	56	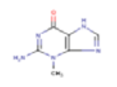	24	13	3
	124	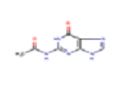	7	8	1
Lumazines	51	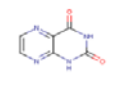	15	8	1
	65	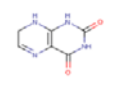	9	12	2
	88	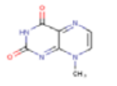	23	9	4
	91	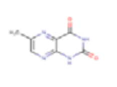	7	8	11
	95	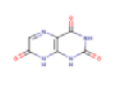	20	5	1
	96	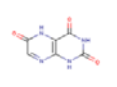	10	11	7

Class	Mol ID	Structure	# of matching peaks	# of missing peaks	# of extra peaks
Lumazines	121	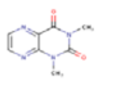	12	2	1
	128	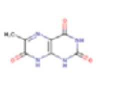	12	7	3
Quinazdine-dion	46	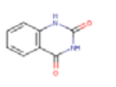	13	10	1
	83	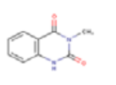	10	1	2
	84	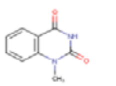	16	10	5
	86	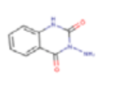	21	12	2
	87	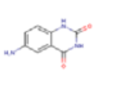	11	7	2
	115	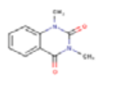	14	1	1
	120	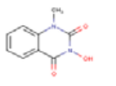	15	3	19
Reumycins	92	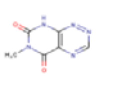	12	18	3
	105	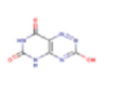	14	13	2
	123	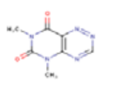	9	4	2
	125	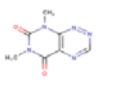	17	20	3
	126	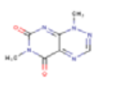	18	11	7
	133	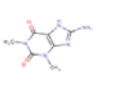	24	7	3
	147	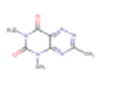	12	6	1
Others 1	47	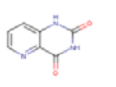	13	8	4

Group	Mol ID	Structure	# of matching peaks	# of missing peaks	# of extra peaks
Others 1	48	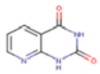	12	3	3
Others 1	50	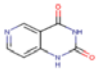	21	13	5
Others 1	116	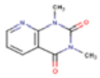	19	7	16
Others 1	117	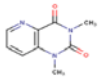	8	1	9
Others 1	118	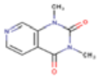	21	9	6
Others 2	38	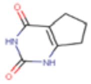	20	17	3
Others 2	70	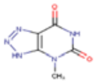	6	3	5
Others 2	74	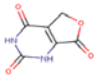	13	12	3
Others 2	80	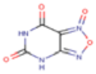	11	7	3
Others 2	93	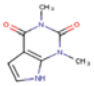	26	14	4
Others 2	122	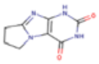	18	7	3
Others 2	146	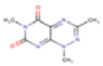	28	12	4
	Total		1162	663	269
